# Development of the mechanoresponsive pericellular matrix of chondrons

**DOI:** 10.1126/sciadv.ado6644

**Published:** 2025-05-02

**Authors:** Donghee Lee, Sydney E. Greer, Andrew T. Dudley

**Affiliations:** Department of Genetics, Cell Biology and Anatomy and Mary and Dick Holland Regenerative Medicine Program, University of Nebraska Medical Center, Omaha, NE 68198, USA.

## Abstract

Physical properties of cartilage are conferred by the composition and ultrastructure of the extracellular matrix. This study focuses on the development of the pericellular matrix (PCM), a domain that directly contacts the chondrocyte and is a key regulator of biomechanical and biochemical signaling. Using three-dimensional cell culture, microfluidic cell compression platforms, and genetic mouse models, we demonstrated that collagen VI is initially assembled at the cell surface and then displaced to form a shell at the PCM-territorial matrix boundary. Cell surface–bound hyaluronan is crucial for the assembly process, and hyaluronan-aggrecan complexes drive displacement. Integrin adhesion is not required early but is crucial to determine the final placement of the collagen VI shell. Dynamic compression accelerated PCM maturation except in aggrecan mutants. Together, these findings provide key insights into the development of the mechanosensitive PCM and establish an in vitro platform to support studies of matrix biology in normal and disease models.

## Teaser

Developmental mechanisms used by chondrocytes generate the mechanoresponsive pericellular matrix.

## INTRODUCTION

The major connective tissues of the musculoskeletal system, including tendons, ligaments, cartilage, and bone, play a vital role in providing structural support and guiding developmental processes (*1*). These tissues are characterized by an extensive extracellular matrix (ECM) composed of interconnected protein fibers, proteoglycans, and glycosaminoglycans (GAGs), which create a three-dimensional (3D) environment supporting cell maintenance, growth, and differentiation (*1*, *2*). Genetic or acquired connective tissue disorders that alter the structural components and affect ECM homeostasis present substantial challenges to human health (*1*, *3*, *4*); however, remaining gaps in knowledge about the relationship between the process of ECM generation, resulting in molecular architecture, and physical properties of the matrix present major obstacles to the development of therapies and regenerative medicine approaches to treating connective tissue diseases.

**Fig. 1.**
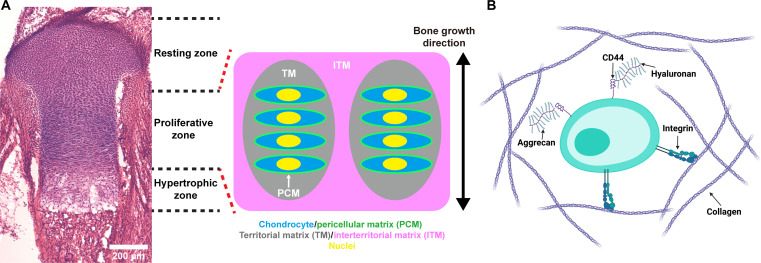
Matrix structure of growth plate cartilage. (**A**) Hematoxylin and eosin staining of a growth plate cartilage in a hindlimb proximal tibia and schematic of matrix architecture of growth plate. Chondrocytes are encapsulated by PCM, and chondrocyte columns are surrounded by a territorial matrix (TM). The chondrocyte columns are separated by interterritorial matrix (ITM). (**B**) Schematic of matrix molecules around a chondrocyte. Collagens attach to the chondrocyte via integrin receptors. HA binds to the cell surface through the CD44 receptor. The CD44 receptor is a cell surface glycoprotein that serves as a receptor for HA, allowing HA to interact with the cell membrane. In addition, aggrecan proteins covalently bind to HA through a link protein.

Cartilage, a connective tissue, boasts a complex, multiterritory ECM structure, serving dual roles: providing crucial structural support and regulating chondrocyte metabolism indispensably. Chondrocytes are surrounded by the pericellular matrix (PCM), which is rich in hyaluronan (HA), the proteoglycan aggrecan, and the nonfibrillar collagen type VI. Together, the chondrocyte and the PCM form the chondron, which represents the primary structural, functional, and metabolic unit of cartilage (*5*). The fibrillar collagen type II–rich territorial matrix surrounds each chondron. Generation of these two territories is under direct metabolic control of the chondrocyte (*6*). The interterritorial matrix, consisting of larger collagen type II fibrils, connects the territorial matrices of adjacent chondrocytes and is viewed as a non–cell-autonomous event in matrix formation (Fig. 1, A and B, and fig. S1) (*7*).

To investigate cell-autonomous mechanisms in ECM formation, it is essential to examine the microlevel spatial organization of the ECM and consider cell-to-cell variations. Historically, the formation of cartilage ECM has been investigated using bulk biochemical testing and measurements, which have limited our understanding of how individual chondrocytes deposit and assemble matrix molecules to create a specific ECM structure (Fig. 1A). However, native cartilage exhibits a highly organized architecture with variations in molecular composition, fibril diameter, and organization, making it difficult to assess these features individually through bulk biochemical assay methods using explant cultures (*2*).

ECM development is also influenced by dynamic mechanical forces transmitted through the ECM to the chondrocytes. Evidence suggests that mechanical stimuli play a critical role in tissue homeostasis and that changes in cell or matrix mechanics can lead to tissue dysfunction and disease by altering matrix secretion and matrix proteinase activity (*8*, *9*). The impact of mechanical stimuli on cells is influenced by various factors, including the magnitude of force, the composition and properties of the ECM through which the stress is transmitted, and the signal transduction from adhesion complexes and other signaling networks involving the cytoskeleton and downstream intracellular pathways (*9*, *10*). Characterizing the effects of compressive stress on the synthesis of PCM at the single-cell level is essential to create a more physiologically relevant environment for studying the assembly of matrix molecules required to develop engineered explants with native cartilage properties.

To enable a more systematic approach to studying matrix development by chondrocytes, we used engineered platforms combined with 3D cell culture techniques. Specifically, we used alginate cultures produced using the alginate bead generator (*11*, *12*) or in our microfluidic compression device (*13*, *14*) combined with whole-mount immunofluorescence and confocal microscopy to study the spatiotemporal deposition of PCM by chondrocytes. Furthermore, we manipulated matrix assembly using specific small molecules and genetic mouse models to provide a comprehensive description of the roles of HA, aggrecan, and integrin β1 (Itgb1) in the stepwise assembly of the PCM (*15*–*17*). In addition, we demonstrated that dynamic mechanical stimulation accelerates PCM development. By adopting this cell-centric approach, we aim to gain a deeper understanding of the intrinsic molecular mechanisms governing matrix secretion and immobilization in the extracellular environment, with the ultimate goal of creating a layered and robust cartilage tissue in cartilage engineering.

## RESULTS

### The PCM territory expands as a function of days of culture

**Fig. 2.**
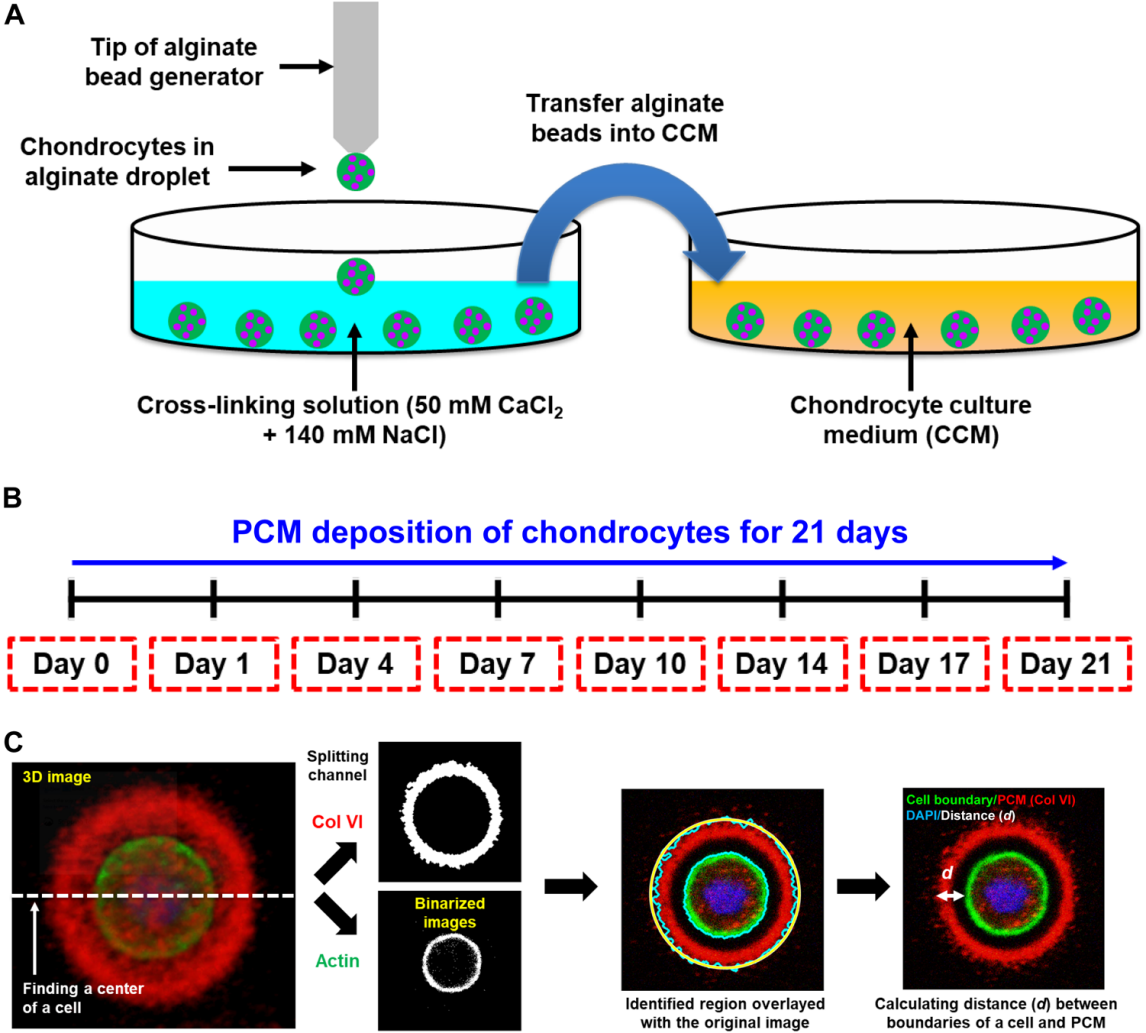
Systems for studying PCM deposition of chondrocytes. (**A**) Chondrocytes were 3D-cultured in alginate beads. A drop of an alginate solution containing chondrocytes was cross-linked in the cross-linking solution and transferred into a chondrocyte culture medium (CCM). (**B**) Chondrocytes were cultured for 21 days to examine the spatiotemporal deposition of chondrocytes. Samples were collected on days 0, 1, 4, 7, 10, 14, 17, and 21 (red dashed boxes). (**C**) Image processing of *z*-stack images of chondrocytes and PCM using a custom-made MATLAB code. The center of a cell was found, and the cross-sectional image of the cell center was used to identify the collagen type VI (Col VI; PCM) and actin (cell boundary). The distance (*d*) between the cell membrane and the PCM boundary was calculated. Blue: 4′,6-diamidino-2-phenylindole (DAPI). Red: Col VI. Green: Actin.

3D cultures of embryonic epiphyseal chondrocytes in 1.5% alginate beads were prepared using a bead generator or a syringe as described (*12*). 3D cell cultures are crucial for maintaining chondrocyte differentiation and preventing dedifferentiation (*11*, *18*). An overview of the procedure and study timeline is provided in Fig. 2. Following fixation, collagen VI was visualized by immunofluorescence, and the cortical actin cytoskeleton was labeled with fluorescent phalloidin to visualize the plasma membrane. Whole-mount specimen preparations were optically sectioned using confocal microscopy. The distance between the cell membrane and the outer boundary of the PCM was calculated from the cross-sectional image at the center of a cell using a custom image processing code developed in MATLAB (Fig. 2C).

**Fig. 3.**
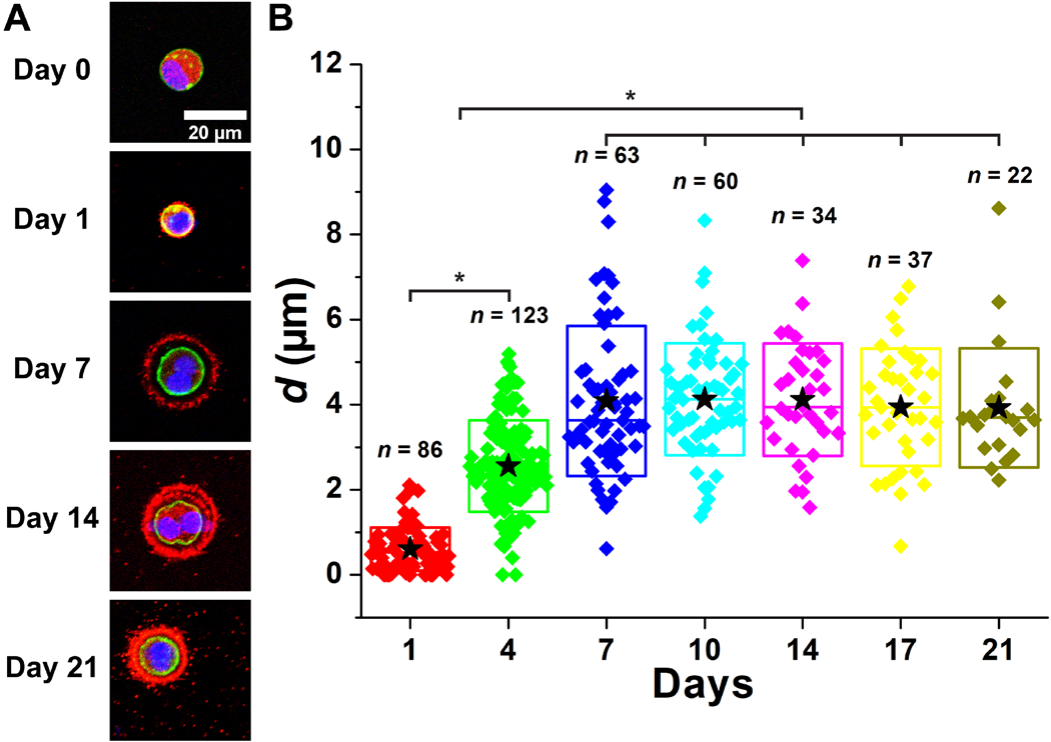
PCM of WT chondrocytes was expanded as a function of days of culture. (**A**) Images of PCM deposition of WT chondrocytes. Blue: DAPI. Red: Col VI. Green: Actin. (**B**) Distance between the cell membrane and the external PCM boundary (*d*) as a function of days of culture. After day 7, mean *d* reached the plateau. *n*: The number of chondrocytes. Star: Mean values. Diamond: Each data point. Top and bottom lines of the box: SD. Middle line of the box: Median value. *P < 0.05.

Figure 3 and fig. S3 illustrate the temporal development of PCM in wild-type (WT) chondrocytes over a 21-day period. On day 0, the collagen VI signal was exclusively intracellular, and no collagen VI was observed around the chondrocytes (Fig. 3A) indicating complete removal of matrix territories following digestion of cartilage with pronase and collagenase. After 24 hours of culture, collagen type VI signal substantially colocalized with phalloidin, demonstrating the presence of collagen VI at the cell membrane. Over the next few days, collagen VI was displaced from the cell membrane to form a shell that progressively extended further away from the cell membrane as the culture duration increased. The distance (*d*) between the cell membrane and the PCM boundary increased with longer culture periods (Fig. 3B). The mean value of *d* reached a plateau of approximately 4 μm on day 7. For this analysis, we excluded multicellular chondrons to specifically observe PCM deposition by a single chondrocyte (or a doublet—daughter cells that have not yet separated) only. However, multicellular chondrons were frequently observed starting around day 4, demonstrating the capability of this in vitro system to generate a key structural hallmark of mature cartilage (fig. S2).

On the basis of these results, an important question is what molecules fill the “empty” space between the cell membrane and the collagen VI shell. The proteoglycans aggrecan and perlecan are previously described as important components of the cartilage matrix, and perlecan has been linked to the mechanoresponsive functions of the PCM (*19*–*21*). Here, we show that at the early stages of PCM development, aggrecan is tightly localized to the cell membrane and the adjacent pericellular space, whereas perlecan is less prominent and primarily localized to a shell that appears associated with collagen VI (fig. S11). Thus, aggrecan is likely to be the major component in the pericellular space during early PCM development.

### Itgb1 is not required to form the collagen VI shell

**Fig. 4.**
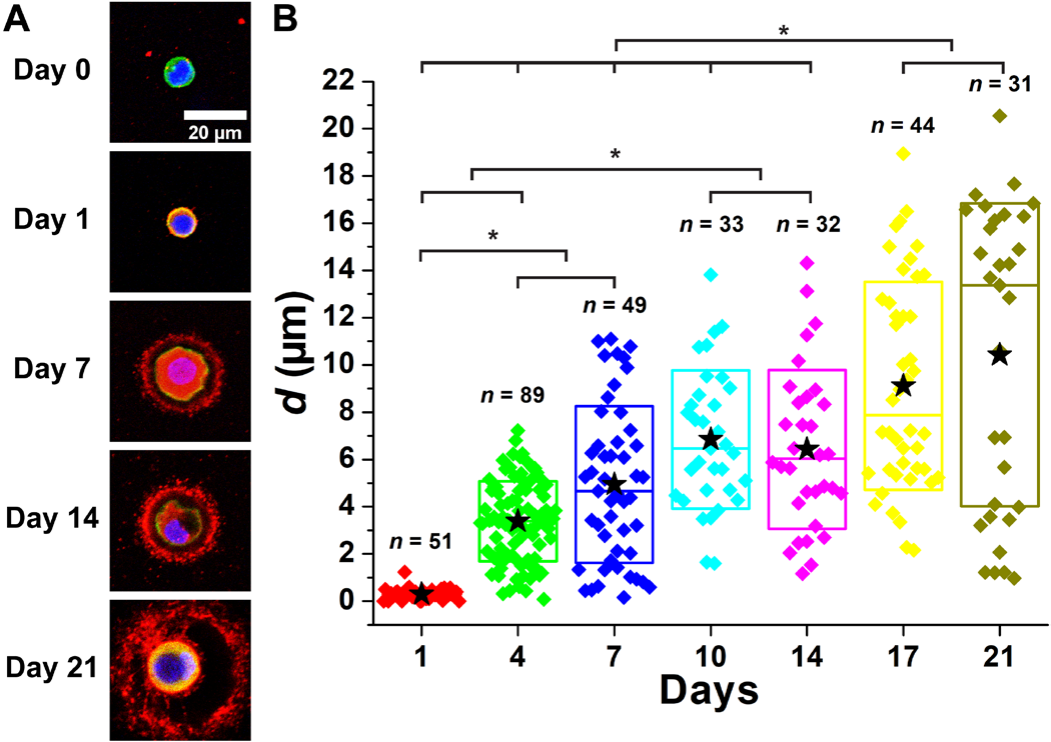
PCM of Itgb1^−/−^ chondrocytes was expanded as a function of days of culture. (**A**) Images of PCM deposition of Itgb1^−/−^ chondrocytes. Blue: DAPI. Red: Col VI. Green: Actin. (**B**) Distance between the cell membrane and the external PCM boundary (*d*) as a function of days of culture. *n*: The number of chondrocytes. Star: Mean values. Diamond: Each data point. Top and bottom lines of the box: SD. Middle line of the box: Median value. *P < 0.05.

Integrins play a crucial role in facilitating the attachment of chondrocytes to the collagenous components of the PCM, allowing for the connection between the intracellular cytoskeleton, the cell signaling proteins, and the ECM. Given the early association of collagen type VI with the cell membrane, we considered whether integrin adhesion might play a role in the assembly of the collagen VI shell. Most integrin heteromeric receptor complexes expressed by chondrocytes contain the Itgb1 subunit. Therefore, we used a tamoxifen-inducible deletion mouse model of Itgb1 in combination with a cartilage-specific, tamoxifen-inducible Col2a1-creERT to determine the effect of loss of integrin adhesion on PCM development. As with WT chondrocytes, we observed the colocalization of collagen type VI with the cell membrane on day 1 and the formation of the collagen VI shell by day 4. However, unlike WT chondrocytes, the *d* of the PCM in Itgb1 mutant (Itgb1^−/−^) chondrocytes did not plateau on day 7 and, instead, progressively increased over the 21 days of culture (Fig. 4 and fig. S4). The mean *d* values for Itgb^−/−^ chondrocyte PCM were larger, and the range of values increased at all time points compared to those of WT chondrocytes. Loss of the *Itgb1* function also does not appear to qualitatively affect the localization of aggrecan or perlecan, which supports the contention that *Itgb1* mutants are not deficient in the early steps of the PCM development process (figs. S10 and S11). Together, these results indicate that Itgb1 is not necessary for assembly or displacement of the collagen type VI shell but may play a role either directly or indirectly in regulating the size of the PCM territory.

### Deletion of aggrecan blocks displacement of collagen type VI from the cell membrane

**Fig. 5.**
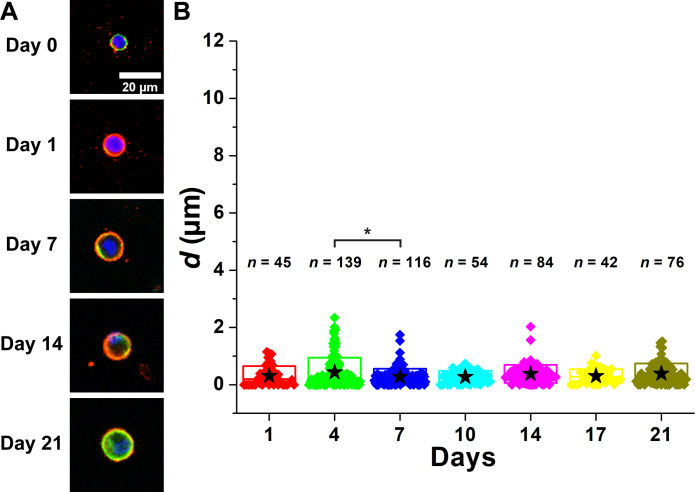
Deletion of aggrecan causes localization of PCM on the membrane of Acan^cmd/cmd^ chondrocytes. (**A**) Images of the PCM deposition of Acan^cmd/cmd^ chondrocytes. Blue: DAPI. Red: Col VI. Green: Actin. (**B**) Distance between the cell membrane and the external PCM boundary (*d*) as a function of days of culture. Mean *d* was less than 1 μm regardless of days of culture. *n*: The number of chondrocytes. Star: Mean values. Diamond: Each data point. Top and bottom lines of the box: SD. Middle line of the box: Median value. *P < 0.05.

We next sought to determine the mechanism that displaces the collagen VI shell from the cell membrane. Previous work by others demonstrated that chondrocytes organize and aggregate complexes of the GAG scaffold HA and the proteoglycan aggrecan (*22*), and we showed the presence of aggrecan in the pericellular space in our cultures (fig. S11). In addition, HA binds directly to cell surface receptors (e.g., CD44), and collagen VI copurifies with HA and aggrecan (*23*, *24*). To test whether aggrecan is required for PCM development, we analyzed the formation of the collagen VI shell in aggrecan-deficient chondrocytes using the constitutive cartilage matrix deficiency mutant mice (Acan^cmd/cmd^). We observed that collagen VI (Fig. 5A and fig. S5) and perlecan (fig. S11) remained localized at the cell membrane of Acan^cmd/cmd^ chondrocytes. The average *d* between the cell membrane and the PCM boundary in Acan^cmd/cmd^ chondrocytes was consistently less than 1 μm throughout the culture period (Fig. 5B). By day 21, some loss of signal at the membrane was observed in aggrecan mutant chondrocytes. However, it is uncertain whether this change reflects internalization and degradation of membrane-associated collagen VI or its release from the cell surface via a receptor down-regulation mechanism. Regardless, these results suggest that the absence of aggrecan led to the failure to generate the PCM compartment, indicating that displacement of the collagen VI shell from the cell membrane is an aggrecan-dependent event.

### Exogenous aggrecan and HA displace collagen VI from the cell membrane

**Fig. 6.**
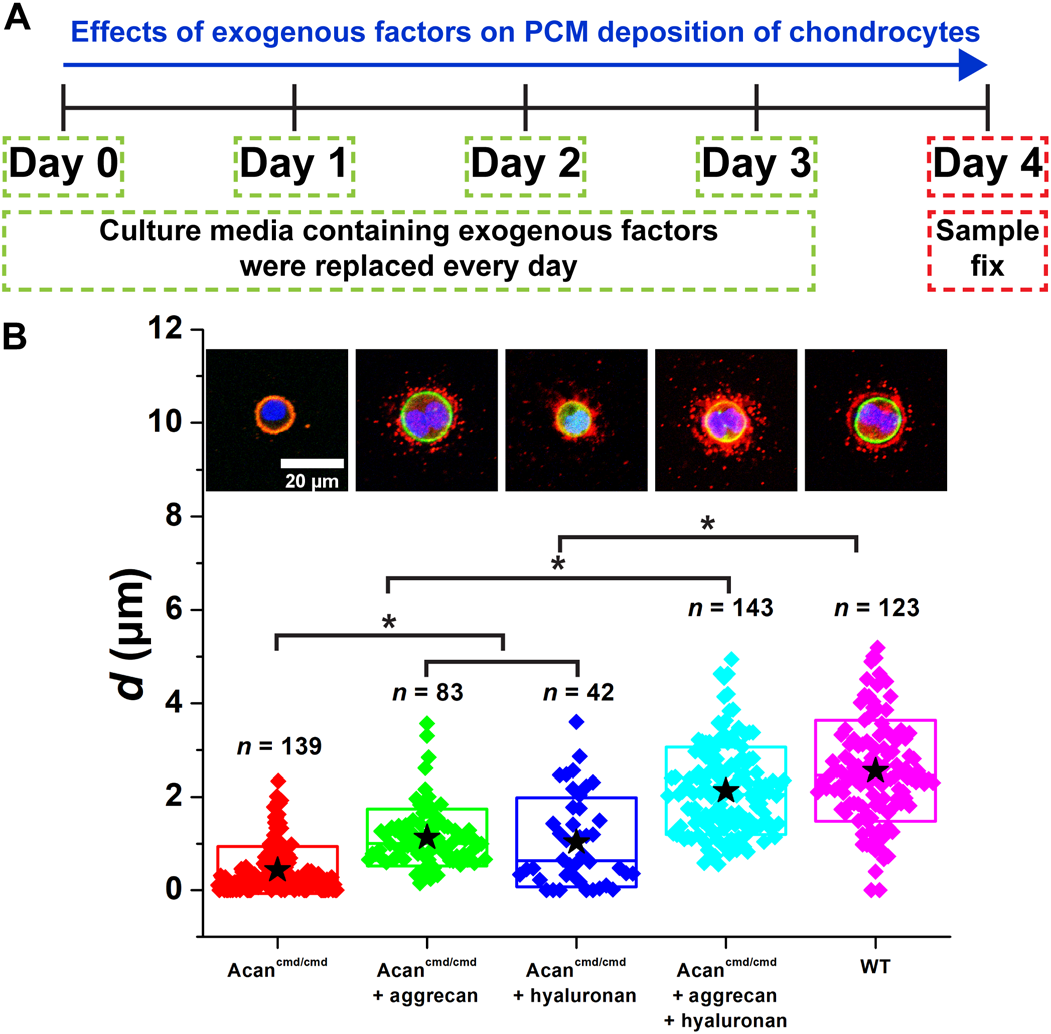
Exogenous aggrecan and HA pushed PCM further from the cell membrane of Acan^cmd/cmd^ chondrocytes. (**A**) Exogenous factors were added to 3D-cultured Acan^cmd/cmd^ chondrocytes from days 0 to 3. On day 4, the sample was collected and fixed. (**B**) The *d* of Acan^cmd/cmd^ chondrocytes with and without the exogenous factors aggrecan and HA. Not only aggrecan but also HA was required to expand the PCM compartment. *n*: The number of chondrocytes. Star: Mean values. Diamond: Each data point. Top and bottom lines of the box: SD. Middle line of the box: Median value. Blue: DAPI. Red: Col VI. Green: Actin. *P < 0.05.

Although the genetic model used suggests that aggrecan specifically is required to displace collagen VI from the cell surface, the potential loss of collagen VI at later culture time points might be consistent with changes in additional molecules or cellular processes. To determine whether aggrecan is the sole missing component in Acan^cmd/cmd^ PCM, we next asked whether the addition of exogenous aggrecan could rescue collagen VI localization (Fig. 6A and fig. S6). When aggrecan alone was added to the culture, a collagen VI–positive shell was observed adjacent to but distinct from the cell membrane (*d* = 1.1 ± 0.6 μm; Fig. 6B). However, rescue was incomplete because the resulting *d* of Acan^cmd/cmd^ chondrocytes treated with aggrecan alone was less than that of WT chondrocytes (*d* = 2.8 ± 1.3 μm) and the collagen VI shell appeared to be discontinuous and mainly composed of individual puncta. In contrast, treatment with HA alone results in qualitatively distinct patches of collagen VI contiguous with the cell surface that might indicate an essential role for HA ultrastructure at the cell surface in the initial assembly of the collagen VI shell (Fig. 6B). We next cultured Acan^cmd/cmd^ chondrocytes with both exogenous aggrecan and HA to test whether the concentration of HA was limiting in this assay. Addition of both HA and aggrecan resulted in a further expansion of the PCM territory (*d* = 2.2 ± 1.0 μm) that more closely resembled the area observed in WT chondrocytes (Fig. 6B). However, with exogenous HA, collagen VI label was observed in a broad domain adjacent to the chondrocyte and colocalized with the cell membrane, and the collagen VI–deficient gap between the cell membrane and the shell was absent as in cultures treated with HA alone. Together, these findings indicate that both aggrecan and HA are important early components for assembling and displacing the collagen VI shell.

### Inhibiting CD44 and HA assembly using HA6 disrupts PCM expansion from the cell membrane

**Fig. 7.**
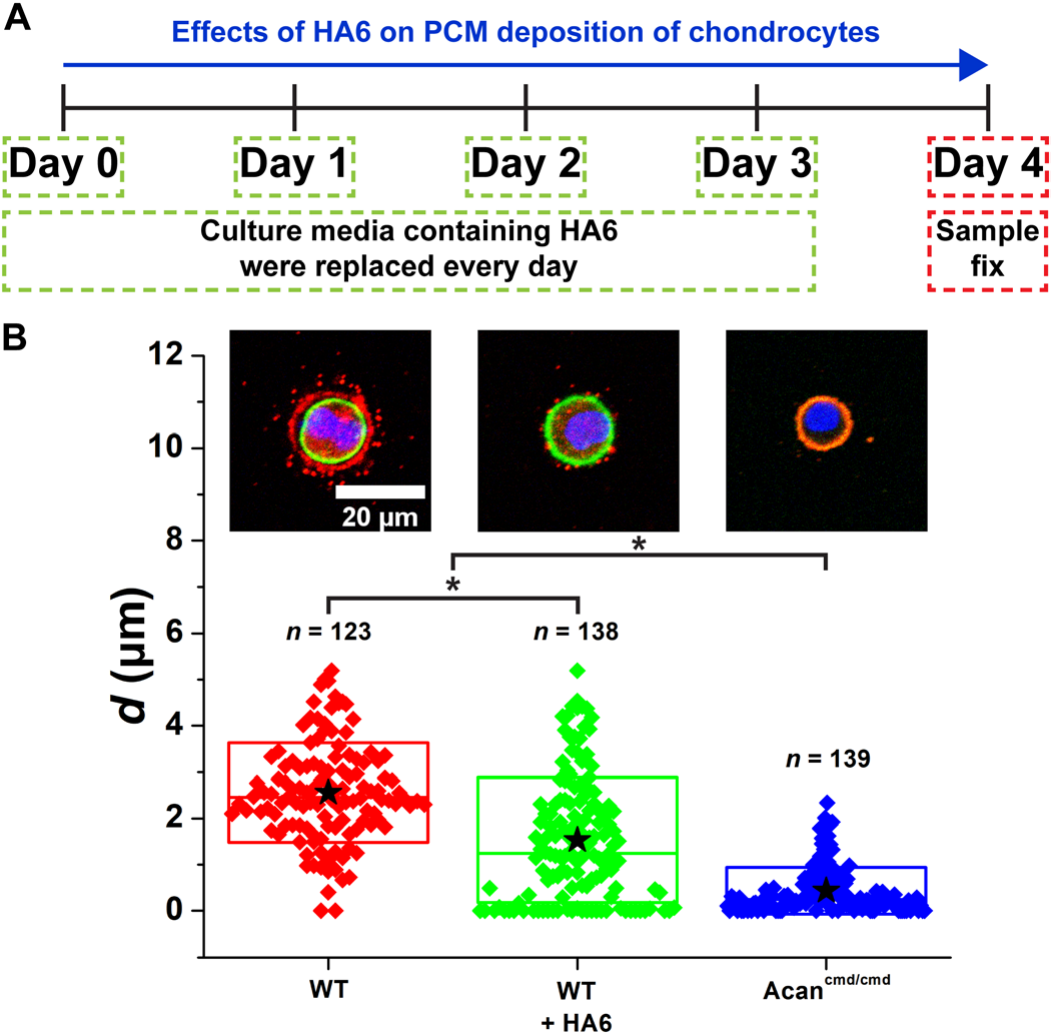
Inhibiting CD44 and HA assembly using HA6 disrupts PCM expansion from the cell membrane. (**A**) 3D-cultured WT chondrocytes were treated with HA6 from days 0 to 3. On day 4, the sample was collected and fixed. (**B**) PCM compartment of HA6-treated WT chondrocytes was reduced. *n*: The number of chondrocytes. Star: Mean values. Diamond: Each data point. Top and bottom lines of the box: SD. Middle line of the box: Median value. Blue: DAPI. Red: Col VI. Green: Actin. *P < 0.05. Data for WT and Acan^cmd/cmd^ chondrocytes are replicated from Fig. 6.

The observations of the partial rescue of the collagen VI shell with aggrecan treatment but substantial disorganization of the shell with addition of HA suggest that HA participates in a saturable process, which is consistent with ligand-receptor interactions. Therefore, we asked whether the adhesion of HA to a cell surface receptor is required for PCM development. Previous studies demonstrated disruption of HA binding to CD44 in chondrocytes following the treatment with CD44 antisense oligonucleotides and small HA fragments (hexa- and octasaccharides) to disrupt the binding of HA to CD44 in chondrocytes (*25*–*27*). We treated chondrocyte cultures with HA hexasaccharide (HA6), which competes with and displaces high–molecular weight HA from CD44 receptors (*7*, *22*, *28*) but does not alter collagen VI or aggrecan expression (fig. S13). Following 4 days of HA6 treatment, the collagen VI shell was absent or reduced to puncta in a substantial number of chondrocytes (Fig. 7). However, unlike in Acan^cmd/cmd^ cultures, collagen VI was absent from the cell membrane following HA6 treatment. Together, these findings suggest that cell-bound HA is essential for generating the collagen VI shell at the outer boundary of the PCM likely by tethering collagen VI to the cell surface at least during the early stages of PCM development.

### Proteoglycan function in displacement of the collagen VI shell

**Fig. 8.**
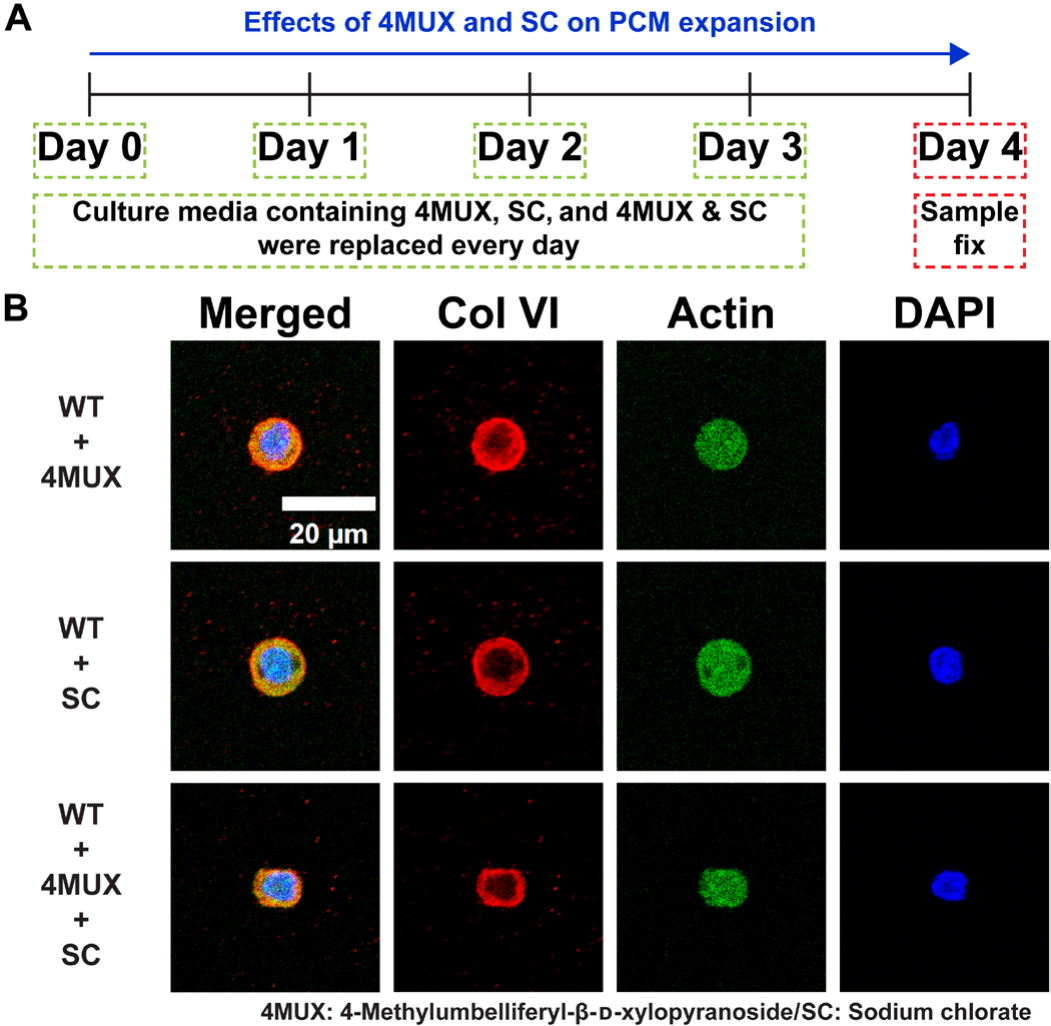
Effects of 4-methylumbelliferyl-β-d-xylopyranoside and sodium chlorate on PCM expansion. (**A**) WT chondrocytes were treated with 4-methylumbelliferyl-β-d-xylopyranoside (4MUX), sodium chlorate (SC), or a combination of 4MUX and SC for 4 days. (**B**) Treatment with 4MUX and SC inhibits PCM expansion on day 4. Blue: DAPI. Red: Col VI. Green: Actin.

The requirement for aggrecan and binding of HA to the cell surface, as well as the presence of perlecan in the early PCM, suggests that proteoglycans might regulate displacement of the collagen VI shell. Proteoglycans contain many GAG chains that display a high negative charge density, which promotes the binding of ions and water (*29*). These hydrated structures have very large hydrodynamic radii, and the swelling of the hydrated molecules can exert high outward forces when constrained in space (e.g., in articular cartilage matrix). To test whether these properties of proteoglycans might affect collagen VI displacement from the cell membrane, we treated cultures with 4-methylumbelliferyl xylopyranoside, which competitively interferes with the initiation of GAG chain addition to proteoglycans (approximately 50% fewer chains) and sodium chlorate, which inhibits sulfotransferases and thereby reduces *O*-sulfation by approximately 70% (*30*–*32*). Expression of collagen VI was unchanged by sodium chlorate treatment, whereas aggrecan expression was approximately 75% of untreated (*P* < 0.05) (fig. S13). These previous studies also showed that inhibitor treatments do not affect total proteoglycan synthesis or secretion from the cell. Treatment with these inhibitors, alone or in combination, resulted in the failure to displace collagen VI from the cell surface, consistent with a requirement for hydration of proteoglycans in this process (Fig. 8 and fig. S8). Removal of the inhibitors led to displacement of collagen VI into the surrounding matrix, but the observed signal was not in a position or of an arrangement consistent with the normal collagen VI shell (fig. S12). These results suggest that disruption of the earliest phase results in unrepairable defects in PCM assembly.

### Compressive stress modulates PCM deposition of WT chondrocytes

**Fig. 9.**
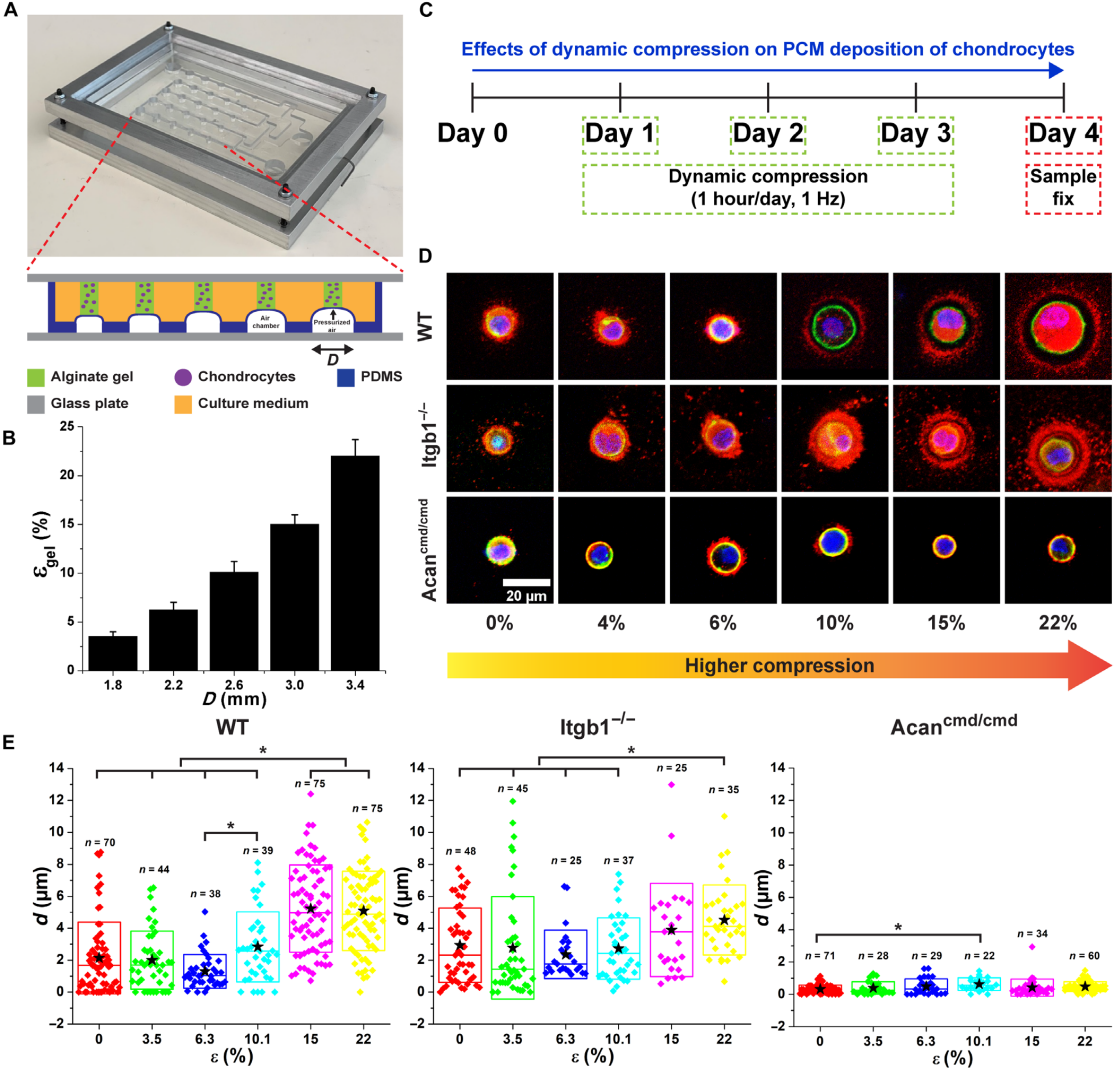
The effects of dynamic compression (1 hour/day, 1 Hz) on PCM deposition of WT, Itgb1^−/−^, and Acan^cmd/cmd^ chondrocytes. (**A**) Cell compression device actuated by pressurized air. The amount of compression was modulated by the diameter (*D*) of air chambers. (**B**) Cell compression device generated compressive strain (ε, 4 to 22%) as a function of the diameter of air chambers. (**C**) Chondrocytes were 3D-cultured in alginate hydrogels and assembled on the device on day 0. The samples were dynamically compressed at 1 Hz for 1 hour on days 1 to 3 and collected on day 4. (**D**) Photos of PCM deposition of WT, Itgb1^−/−^, and Acan^cmd/cmd^ chondrocytes as a function of the magnitudes of compression. Blue: DAPI. Red: Col VI. Green: Actin. (**E**) The *d* of chondrocytes as a function of ε. The effects of compression on *d* were negligible on Acan^cmd/cmd^ chondrocytes as compared to noncompressed samples. *n*: The number of chondrocytes. Star: Mean values. Diamond: Each data point. Top and bottom lines of the box: SD. Middle line of the box: Median value. *P < 0.05.

Compressive stress is a major mechanical force experienced by chondrocytes and is known to influence skeletal growth by modifying the structure and dimensions of the growth plate (*9*, *33*, *34*). To test a range of compressive stress, we generated microfluidic devices that simultaneously generate five different levels of compression (expressed as compressive strain, ε = 4 to 22%) on a single platform by adjusting the diameter (*D*) of the air chambers (Fig. 9, A and B). Further details regarding the cell compression device can be found in our previous publications (*13*, *14*). Chondrocytes were cultured in the compression device for a period of 4 days, and cells were subjected to compression for 1 hour/day at a frequency of 1 Hz, excluding days 0 and 4 (Fig. 9C). Previous investigations of dynamic compression on growth plate cartilage in vivo and ex vivo have reported differences in growth outcomes within the frequency range of 0.1 to 1.0 Hz (*9*). In addition, as little as 15 min of dynamic compressive loading at 1.1 Hz can induce changes in the phosphorylation patterns of cytoskeletal proteins, while a 30-min duration of loading can lead to alterations in the phosphorylation of proteins associated with transcriptional regulation (*35*). Although these studies provide a rationale for the experimental design, the objective of studying dynamic compression is not to replicate physiological activity but rather to characterize the influence of a range of compressive stress levels on matrix assembly and to assess the suitability of our culture model for studying the effects of mechanical forces.

In compression studies, we observed that the *d* of the PCM in WT and Itgb1^−/−^ chondrocytes increased with the magnitude of compression applied (Fig. 9, D and E, and fig. S9). When the compressive strain (ε) was below 10%, the mean *d* of WT and Itgb1^−/−^ chondrocytes was approximately 2 and 3 μm. However, when ε exceeded 15%, WT and Itgb1^−/−^ chondrocytes deposited matrix components further away from the cell membrane. The mean *d* of WT and Itgb1^−/−^ chondrocytes approximately doubled when ε = 22%. Of note, although Itgb1^−/−^ cells responded to compression, the magnitude of response appeared muted compared to WT cells, and collagen VI organization in the PCM and localization to the cell membrane were qualitatively distinct from WT chondrocytes. In contrast, collagen VI remained localized at the cell membrane of Acan^cmd/cmd^ chondrocytes regardless of the applied compressive strain. These findings support a requirement for aggrecan and a role for Itgb1 in generation of the PCM territory.

## DISCUSSION

**Fig. 10.**
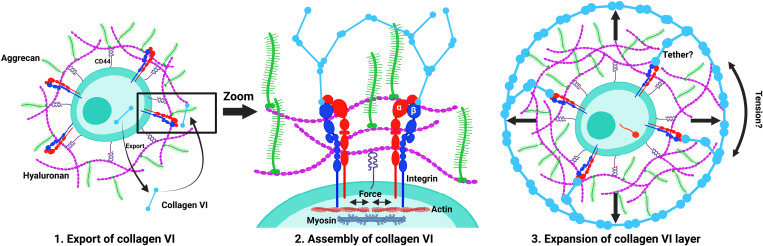
Three-stage process governing the development of the PCM of chondrocytes. The initial stage involves the export of collagen VI to the cell surface, followed by the assembly of collagen VI into a structured layer. Notably, high–molecular weight HA at the cell surface plays a crucial role in this assembly. The final stage encompasses the expansion of the collagen VI layer, facilitated by aggrecan. This expansion results in the formation of a gap between the cell membrane and the collagen VI shell, likely filled with HA-aggrecan complexes. Created with BioRender.com.

Decades of research have identified the key components of the cartilage ECM and elucidated the function of each component. However, despite this wealth of knowledge, therapeutic approaches that restore function to diseased ECM and engineered cartilage that exhibits the mechanical properties of native tissue are lacking. One challenge in defining relationships between ECM components, matrix architecture, and mechanical properties of tissue is the current reliance on in vivo models in which matrix formation cannot be easily observed in real time and the contribution of newly synthesized matrix molecules cannot be distinguished from preexisting molecules. In addition, confounds in existing in vivo models complicate the determination of direct versus indirect mechanisms when deletion of one component leads to structural changes in other components. Moreover, it is difficult to use in vivo models to analyze heterogeneity in ECM production and mechanical responsiveness at the cellular level and to assess the individual contributions of neighboring cells to the local matrix microenvironment. Here, we describe the development and application of an in vitro model of cartilage ECM development that is amenable to chemical and genetic manipulation, accessible for biochemical and molecular analysis of ECM production in single cells, and compatible with force-generating microfluidic devices to enable studies of interactions of matrix architecture and mechanobiology of cartilage. The focus of these studies was on the deposition of the PCM protein collagen VI, as the PCM is the initial matrix produced by chondrocytes and plays a crucial role in mediating chondrocyte response to biochemical and biomechanical signals (*36*). These studies revealed a three-stage process of PCM development that includes export to the cell surface, HA-dependent assembly at the cell surface, aggrecan-dependent “inflation” of the collagen VI shell, and important roles for Itgb1-mediated adhesion and compressive forces in accelerating PCM development (Fig. 10).

The first two stages of PCM development involve intracellular trafficking of collagen VI and assembly on the cell surface. During these stages, export of collagen VI, aggrecan, and perlecan to the cell surface was unaffected by mutations in *Acan* and *Itgb1* as indicated by colocalization of immunofluorescence signal with actin. These observations are both expected, because these molecules interact with collagen VI in the extracellular space and are informative, as they show that the mutant loci do not produce aberrant proteins that substantially interfere with protein trafficking and loss of these adhesion interactions does not result in signaling feedback that meaningfully alters cell processes, such as survival or protein synthesis. Only treatment with HA6 oligomers that release high–molecular weight HA from cell surface receptors blocked the accumulation of collagen VI at the cell surface, indicating that cell surface–associated HA rather than bulk HA is crucial for the initial assembly process (*23*). It is not clear at this time whether assembly is exclusively mediated by the CD44 receptor (*37*, *38*) because HA6 has only been demonstrated to interfere with HA binding to CD44 (*7*) but CD44 mutants show compensation by RHAMM (receptor for HA-mediated motility), an alternative HA receptor (*39*), suggesting that potential roles for or compensation by other HA receptors remains possible. Although these findings support the conclusions from other groups (*22*), normal cell surface localization of collagen VI on *Acan^cmd/cmd^* mutant chondrocytes demonstrates that collagen VI enrichment at the cell membrane is mostly dependent on HA and not the HA-associated proteoglycan aggrecan. The observation that exogenous HA is permissive to PCM development but leads to disorder in the collagen VI shell further differentiates this model from previous results by showing that the structure of HA physically linked to the cell surface, and not just the presence of HA in the microenvironment, is essential for normal ECM development. We postulate that endogenous HA and aggrecan are assembled in a “planar” layer at the cell surface onto which collagen VI is layered. In contrast, free HA saturates the cell surface receptors and leads to a different, nonplanar arrangement of HA and aggrecan that allows incomplete assembly both on and beyond the cell surface but precludes displacement by the mechanism discussed below. This is not likely due to an imbalance in the ratio of HA and aggrecan because treatment with both presents the same defect although the domain of collagen VI distribution is enlarged.

The third stage of development involves displacement of the assembled collagen VI fibrils from the cell surface. Displacement of collagen VI is dependent on aggrecan because exogenous aggrecan rescues displacement in *Acan^cmd/cmd^* chondrocytes. Collagen VI is displaced as an intact shell that likely contains perlecan with the gap between the cell membrane and the shell lacking or containing a low concentration of collagen VI as indicated by the absence of signal in immunofluorescence. The space devoid of collagen VI is filled with complexes of HA and aggrecan assembled at the cell surface. Expansion of the pericellular space and consequent displacement of the collagen VI shell are dependent on proteoglycan function as demonstrated by two treatments that affect GAG function via distinct mechanisms. However, as both inhibitors potentially affect the hydrodynamic radius and swelling of the HA-aggrecan complexes, we cannot, at this time, distinguish between a mass-driven displacement and an osmotic inflation mechanism. Future measurement of forces acting on the pericellular capsule will resolve this uncertainty.

One potential caveat to this interpretation derives from an incomplete understanding of the mechanism by which exogenous HA and aggrecan promote inflation of the collagen VI ring. The concern is about how these large molecules penetrate the collagen VI ring and become concentrated at the cell surface to promote inflation. Attempts to visualize the localization of exogenously added molecules have been unsuccessful due to low signal-to-noise ratio. However, the image data provide circumstantial evidence in support of our model. In the absence of aggrecan (*Acan* mutant cells), collagen VI (red) remains colocalized with the cell membrane (green; resulting in yellow in regions of overlap). This observation is consistent with the idea that HA present at the cell surface binds to and enables assembly of collagen VI but lacks the ability to displace the pericellular capsule. Exogenous aggrecan alone, likely acting in concert with endogenous HA enriched at the cell membrane, is sufficient to displace collagen VI, as demonstrated by the lack of colocalization. While the displacement does not achieve the same extent as in WT cells, the resulting collagen VI ring is more ordered and closely resembles WT structures. In contrast, all other treatments resulted in disorder of collagen VI, suggesting that exogenous HA competes with the endogenous HA binding of collagen VI, resulting in a “dominant-negative” phenotype of displacement with disorder and incomplete removal from the cell surface.

For conditions with exogenous HA or HA + aggrecan, the data show that these components are capable of binding and aggregating collagen VI. However, the lack of structural organization or proper cell surface localization of exogenous molecules appears to interfere with normal PCM displacement. This interference leads to the incomplete removal of collagen VI from the cell surface, as observed in our experiments.

Our studies show the stepwise assembly of major matrix components that comprise the PCM territory. However, the early localization patterns described here do not precisely mimic the latter patterns in more mature cartilage. For example, in cartilage, perlecan has been localized adjacent to the cell (likely PCM) but also shown throughout the territorial and interterritorial matrices (*19*, *20*, *40*). These differences could relate to differences in in vitro and in vivo models or to differences in protein secretion and matrix assembly at different stages of development. Alternatively, these differences could derive sequentially from the same assembly mechanisms. One interpretation of the results is that binding sites on the cell surface or matrix molecules are limited and therefore saturable. In this case, the initial interactions would favor the available higher-affinity binding sites, while after saturation of these sites, additional protein molecules could exhibit different interactions and localization within the matrix.

In recent years, there has been growing evidence implicating mechanical forces in the regulation of cartilage formation and growth in vivo. In particular, the effects of dynamic or static compression on skeletal growth and the architecture of the growth plate have been extensively studied (*41*–*43*). In certain models, the absence of compression resulting from limb paralysis has been shown to lead to reduced growth and alterations in the structure of the growth plate (*44*). However, investigating how compression influences microlevel chondrocyte functions such as matrix deposition and remodeling is challenging in in vivo models, primarily due to the lack of control over physiological factors and limitations in monitoring single-cell–level behaviors. In these studies, we show that dynamic compression accelerates matrix development such that higher strain leads to more rapid maturation of the PCM without increasing the size of the PCM domain. Acceleration of matrix development is also observed in *Itgb1* mutant chondrocytes, suggesting that either mechanotransduction is Itgb1 independent or other cell-matrix interactions compensate in *Itgb1* mutants.

One important question that remains unanswered by these studies is the mechanism that limits the extent of displacement of the shell from the cell surface in WT chondrocytes. Synthesis of HA and aggrecan could, in theory, determine the displacement, but the feedback systems that down-regulate synthesis after displacement are not known. Our initial hypothesis that collagen VI tethers connect the shell to cell surface integrins could provide both a physical constraint (length of the tether) and a signaling feedback system (i.e., tension on integrin complexes). This model is supported by the failure to restrain collagen VI displacement in *Itgb1* mutant chondrocytes but is not consistent with the absence of detectable collagen VI in the gap; although dispersed, thin collagen VI tethers might not be detected by immunofluorescence. Perlecan is another Itgb1-interacting molecule that connects multiple matrix molecules including collagens and thus could function as a tether (*45*). However, as with collagen VI, we did not observe perlecan localization to the pericellular space at the early stages of PCM development. An alternative model for regulation of the size of the PCM domain is that the shell is fully assembled on the cell surface and inflation of the proteoglycan compartment continues until the collagen VI layer achieves maximum stretch, which could integrate perlecan localization and function as well. In this model, tension may be essential to the integrity of the shell. If so, then the *Itgb1* phenotype could be explained if cell-matrix adhesion imparts pretension at the cell surface to facilitate assembly. Consistent with this model, the loss of shell integrity during continued expansion in *Itgb1* mutants may reflect the failure of this early pretension step. A potential role for integrin function in shell assembly is also supported by the observation that some collagen VI is retained at the cell surface in *Itgb1* mutants (both static and dynamic compression cultures) but not in WT chondrocytes. However, it is not clear whether this observation indicates the requirement for direct integrin-collagen VI interaction or reflects indirect effects from the incorrect assembly of other matrix molecules, such as fibronectin, for which tension is a regulator of structure (*46*–*48*).

The studies described here focus on the assembly of the PCM, one component of the cartilage matrix. We present an in vitro system to analyze matrix development by chondrocytes and demonstrate that the use of genetic mutants, chemical inhibitors, and exogenous protein treatments can be used to dissect mechanisms of matrix assembly. We chose to focus the initial studies on the PCM that is crucial to the mechanobiology of chondrocytes but appreciate that the overall structural integrity of the cartilage matrix requires the collagen II–rich territorial and interterritorial matrix domains. The territorial and interterritorial matrix domains are distinguished, in part, by a more disordered fiber orientation in the former and thicker, aligned collagen fibers in the latter. It will be interesting to evaluate the mechanisms of territorial and interterritorial matrix formation and to determine whether the generation of these domains depends on the process of PCM assembly.

## MATERIALS AND METHODS

### Mouse genetics and tamoxifen injections

For all matings, embryonic day 0.5 (E0.5) was designated as noon on the day of the postcoital plug. Acan^cmd/cmd^ mice were generated by breeding Acan^cmd/+^ mice with each other (Acan^cmd/NKruJ^, the Jackson Laboratory, stock no. 010522), as previously described (*15*, *17*). Itgb1^−/−^ mice were generated by breeding Itgb1^fl/fl^ (B6;129-Itgb1tm1Efu/J, the Jackson Laboratory, stock no. 004605) and Col2a1-CreERT mice [FVB-Tg (Col2a1-cre/ERT)KA3Smac/J, the Jackson Laboratory, stock no. 006774], as previously described (*16*). For the induction of conditional mutations, pregnant females were injected intraperitoneally on E12.5, E13.5, and E14.5 with a dose (100 mg/kg) of tamoxifen and a dose (50 mg/kg) of progesterone [tamoxifen (20 mg/ml), progesterone (10 mg/ml) in 90% corn oil, and 10% ethanol] to generate a pulse of cre activity and induce recombination in the floxed alleles, resulting in the creation of Itgb1^−/−^ genotype. WT mice were generated using Acan^+/+^ mice. All procedures were conducted in accordance with the regulatory agency policies and approved by the Institutional Animals Care and Use Committee (protocol no. 18-038) at the University of Nebraska Medical Center.

### Histology

Mice were euthanized, and the knee joints were harvested at E17.5. For histology, samples were fixed in 4% paraformaldehyde (PFA) in phosphate-buffered saline (PBS; pH 7.4). After 24 hours of fixation, samples were washed three times with PBS for 5 min each. Subsequently, samples were treated with 30% sucrose overnight and then placed in a mixture of 30% sucrose and optimal cutting temperature (OCT) compound (1:1 volume ratio) overnight. Samples were embedded in OCT and frozen at −80°C. Nonsequential 14-μm sections were collected on each microscope slide from each specimen and stained with hematoxylin and eosin for general histology. Comparable sections of the proximal tibial growth plate were chosen on the basis of the anatomical structures of the joint.

### Chondrocyte isolation

Primary murine growth plate chondrocytes were harvested from the growth plate cartilages of the hind limbs at E17.5. Cells were collected from a total of 15 embryos, which were sourced from two to three different mothers to ensure genetic variability and minimize litter-specific effects. The cartilages were sequentially digested by incubating them in 1% (w/v) pronase for 30 min, followed by 0.25% (w/v) collagenase for 3 hours at 37°C. This digestion process allowed for the release of chondrocytes from the ECM of the cartilage. Subsequently, the chondrocytes were pelleted by centrifugation at 150*g* for 5 min, and the supernatant was carefully removed. The chondrocyte pellet was then resuspended in Dulbecco’s modified Eagle’s medium containing 0.075% (w/v) bovine serum albumin (BSA). After another round of centrifugation at 150*g* for 5 min, the chondrocyte pellet was resuspended in a 1.5% (w/v) alginate (Pronova UP MVG, FMC Corporation, Philadelphia, PA) gel solution, which was filter sterilized and prepared using PBS. Experiments were conducted in two to three independent biological replicates to ensure reproducibility of results and minimize experimental variability.

### 3D chondrocyte culture in alginate beads and cell compression device

Primary chondrocytes were cultured in a 3D environment using spherical droplets of 1.5% (w/v) alginate solution. The chondrocytes were suspended at a concentration of 3.0 × 10^6^ cells/ml in the alginate solution. These droplets were then carefully added to a cross-linking solution composed of 50 mM calcium chloride (CaCl_2_) and 140 mM NaCl. The cross-linking solution facilitates the formation of solid alginate beads. Our laboratory has previously demonstrated that 1.5% (w/v) alginate is suitable for supporting chondrocyte viability, as well as promoting their differentiation and proliferation (*11*, *12*). The alginate beads were generated using a 3D-printed alginate bead generator, which uses airflow to detach the beads from a continuous stream of hydrogel solution (*12*). Subsequently, the alginate beads were cultured in chondrocyte culture media (CCM) following the protocol described by Erickson *et al.* (*11*) for a period of 21 days. At specific time points (days 0, 1, 4, 7, 10, 14, 17, and 21), the alginate bead cultures were fixed with 4% PFA in tris-buffered saline (TBS) containing 5 mM CaCl_2_. The fixed samples were then stored at 4°C for further analysis.

To investigate the impact of mechanical stress on PCM deposition of chondrocytes, we developed a custom cell compression device (Fig. 8A) capable of applying controlled compressive stress to chondrocytes cultured in an alginate scaffold 3D system. The fabrication and validation of this device were previously described in detail in our publications (*13*, *14*). The cell compression device was created using polydimethylsiloxane through a standard soft lithography technique. It features a 5 × 5 array of alginate-chondrocyte constructs, allowing us to simultaneously apply five different magnitudes of compression (compressive strain, ε = 3.5, 6.3, 10.1, 15.0, and 22.0%) with five technical replicates for each level of compression using a single device. In our experimental setup, the chondrocytes within the cell compression device underwent compression at a frequency of 1 Hz for 1 hour/day. This compression regimen was performed daily from days 1 to 3, excluding days 0 and 4. On day 4, the chondrocytes were fixed using 4% PFA in TBS containing 5 mM CaCl_2_ for subsequent analysis and evaluation of PCM deposition. This cell compression device provides a controlled mechanical stimulation platform to investigate the effects of compressive stress on chondrocyte behavior and PCM deposition in our alginate-based 3D culture system.

### Testing exogenous factors

To investigate the effects of exogenous aggrecan and HA on PCM deposition, 3D-cultured Acan^cmd/cmd^ chondrocytes were treated with the CCM supplemented with either exogenous aggrecan (A1960-1MG, Sigma-Aldrich, St. Louis, MO) or a combination of exogenous aggrecan and HA (>950 kDa; GLR002, R&D Systems, Minneapolis, MN) for a duration of 4 days (Fig. 6A). Aggrecan and HA were each used at a concentration of 0.25 mg/ml. To disrupt the binding of high–molecular weight HA to CD44, 3D-cultured WT chondrocytes were treated with HA6 in the CCM for 4 days (Fig. 7A and fig. S7). The concentration of HA6 used was 0.25 mg/ml, according to previous studies (*22*). GAG synthesis was inhibited with 1 mM 4-methylumbelliferyl xylopyranoside (A7008, Sigma-Aldrich, St. Louis, MO) and/or 50 mM sodium chlorate (403016, Sigma-Aldrich, St. Louis, MO) in the CCM. At the end of the treatment period, the chondrocyte bead cultures were fixed using 4% PFA in TBS containing 5 mM CaCl_2_ for subsequent analysis and evaluation.

### Whole-mount immunofluorescence staining

The fixed bead and compression samples were subjected to a series of washing steps. First, they were washed twice with TBS containing 5 mM CaCl_2_ for 5 min at room temperature. Following the washes, the samples were permeated with a polyacrylamide solution composed of 4% acrylamide and 0.13% bis-acrylamide (Bio-Rad Laboratories, Hercules, CA) in TBS containing 5 mM CaCl_2_ at 4°C overnight. The polyacrylamide solution was then removed from the sample container. To initiate the cross-linking of the polyacrylamide gel, a fresh polyacrylamide solution containing ^1^/_100_ total volume of ammonium persulfate [10% (w/v)] and ^1^/_1000_ total volume of tetramethylethylenediamine was injected into the sample container. This initiated the cross-linking process. The samples were isolated from the bulk polyacrylamide gel and subsequently blocked with 5% BSA in PBS with 0.1% Triton X-100 (PBST) for a duration of 1 day. Next, the samples were incubated with rabbit anti–collagen VI antibody (1:125; Abcam, Cambridge, UK) in 1% BSA in 0.1% PBST for 2 days. After incubation, the samples were washed for 1 day with 1% BSA in 0.1% PBST. Subsequently, the samples were incubated with a secondary anti-rabbit Alexa Fluor 555 antibody (1:125; Cell Signaling Technology, Danvers, MA) and phalloidin-labeled Alexa Fluor 488 (1:200; Invitrogen, Waltham, MA) for 2 days in 1% BSA in 0.1% PBST. Before mounting, the samples were incubated with 300 μM DAPI (4′,6-diamidino-2-phenylindole) in 1% BSA in 0.1% PBST for 3 hours, followed by an overnight wash in 1% BSA in 0.1% PBST. The samples were then placed on a glass-bottom petri dish (LabTek, Grand Rapids, MI), mounted in PBS, and held in place by a glass coverslip. The *z*-stack images of PCM and chondrocytes were obtained by confocal microscopy with a *z*-step of 1 μm using a 25× water immersion lens (ZEISS 880, Carl Zeiss AG, Oberkochen, Germany). To analyze the images, a custom batch processing analysis was developed in MATLAB. This analysis used optical sections to determine the center of each cell and create binary images (Fig. 2C). These binary images were then used to calculate the average *d* between the cell membrane (labeled with phalloidin) and the external boundary of the PCM (labeled with collagen VI).

### Quantitative reverse transcription polymerase chain reaction

Total RNA was isolated from three alginate beads using NucleoSpin column isolation (Takara). All other reagents and instruments were supplied by Thermo Fisher Scientific. RNA was digested with deoxyribonuclease I, oligo(dT)_20_ (50 μM) and random hexamers (50 ng/μl) were annealed, and cDNA was synthesized using SuperScript IV as per the manufacturer’s protocol. Relative transcript levels for collagen VI (forward primer: TCAAGAGCCTGCAGTGGATG; reverse primer: TGGACACTTCTTGTCTATGCAG) and aggrecan (forward primer: TCACTGTTACCGCCACTTTCC; reverse primer: TGCTGCTCAGATGTGACTGC) were normalized to β-actin (forward primer: AGCGAGCATCCCCCAAAGTT; reverse primer: GGGCACGAAGGCTCATCATT) using 2X ABsolute Blue SYBR Green ROX polymerase chain reaction (PCR) master mix. Nonreverse transcribed control samples were included in the analysis. Reactions were run in duplicate for three biological replicates using an ImageQuant quantitative PCR instrument. Relative expression levels were calculated using the delta-delta comparison method.

### Statistical analysis

The *d* values were reported as the means ± SD. To assess the statistical significance of the calculated mean values between multiple groups, a one-way analysis of variance (ANOVA) was performed. Subsequently, Tukey’s multiple comparisons test was used to compare the means between individual groups. A significance level of 0.05 was chosen for the statistical analysis, indicating that *P* values less than 0.05 were considered statistically significant.
