# Supplementary material for: Development of the mechanoresponsive pericellular matrix of chondrons
Source: Sci Adv. 2025 May 2;11(18):eado6644. doi: 10.1126/sciadv.ado6644 (PMC12047428; doi:10.1126/sciadv.ado6644)
Supplement: Supplementary file 1 — Figs. S1 to S13 Table S1 [file sciadv.ado6644_sm.pdf]

Science Advances
AAAS

# Supplementary Materials for

## Development of the mechanoresponsive pericellular matrix of chondrons

Donghee Lee *et al.*

Corresponding author: Andrew T. Dudley, andrew.dudley@unmc.edu

*Sci. Adv.* **11**, eado6644 (2025)
DOI: 10.1126/sciadv.ado6644

**This PDF file includes:**

Figs. S1 to S13
Table S1

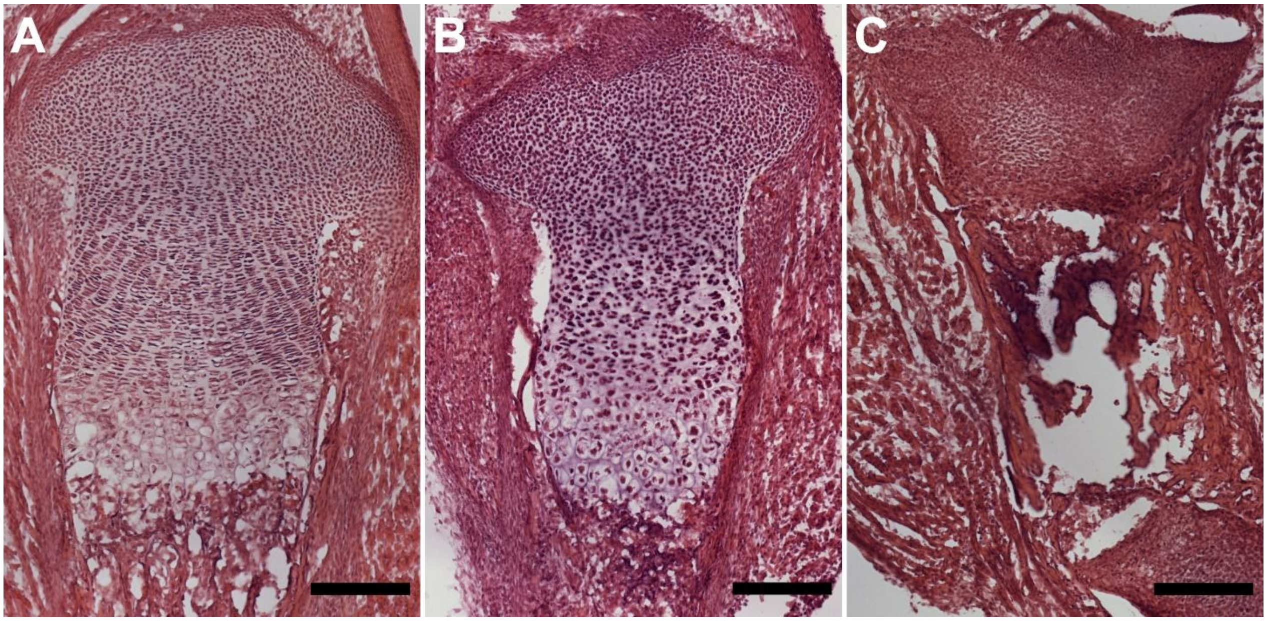

Fig. S1. Hematoxylin and eosin staining of a hindlimb proximal tibia section (E 17.5). (A) WT. (B) Itgb1$^{-/-}$. (C) Acan$^{cmd/cmd}$. Scale bars: 200 μm.

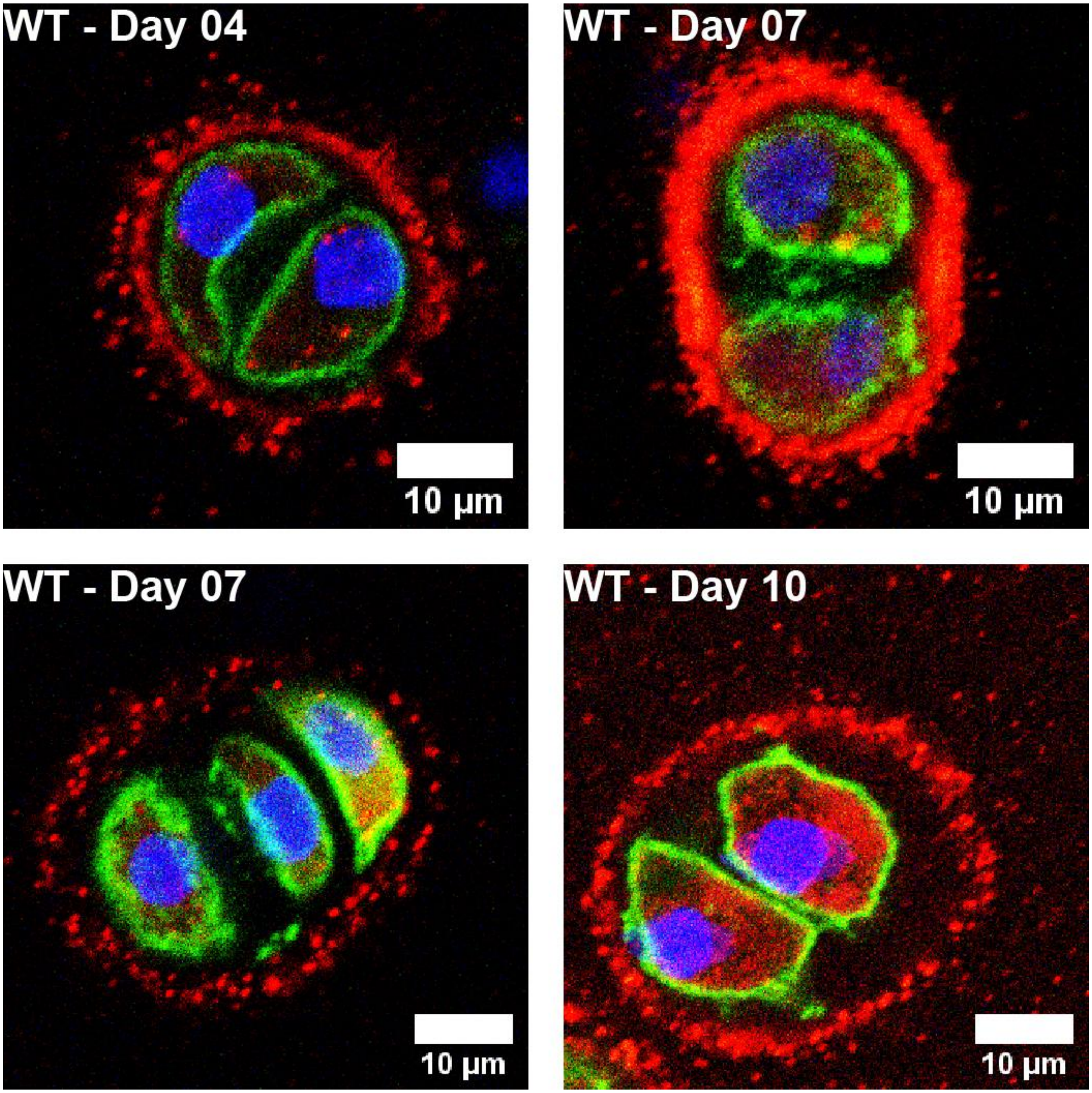

**Fig. S2. Examples of chondrons.** Blue: DAPI. Red: Col VI. Green: actin.

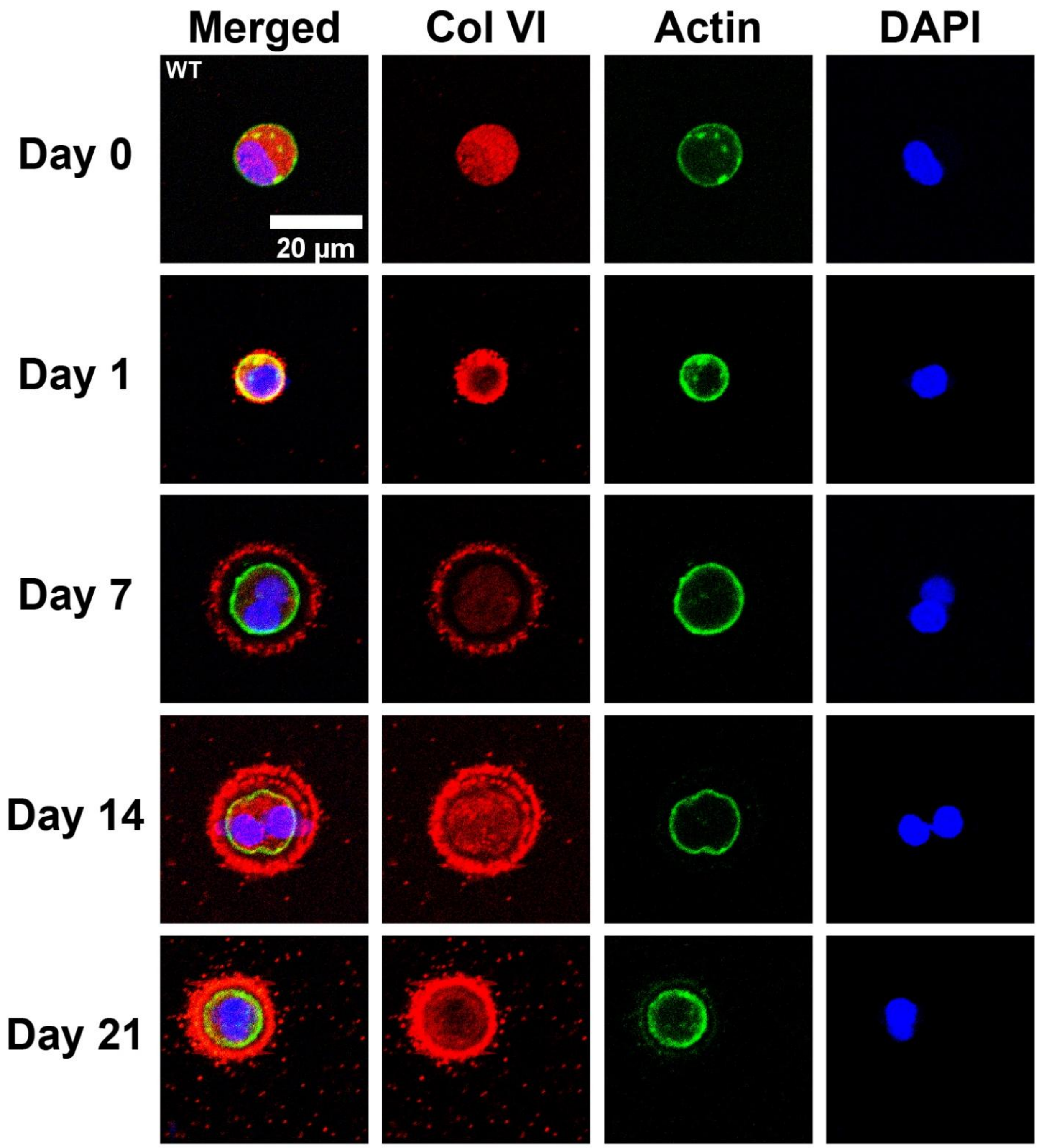


**Fig. S3. PCM of WT chondrocytes was expanded as a function of days of culture**. Blue: DAPI. Red: Col VI. Green: actin.

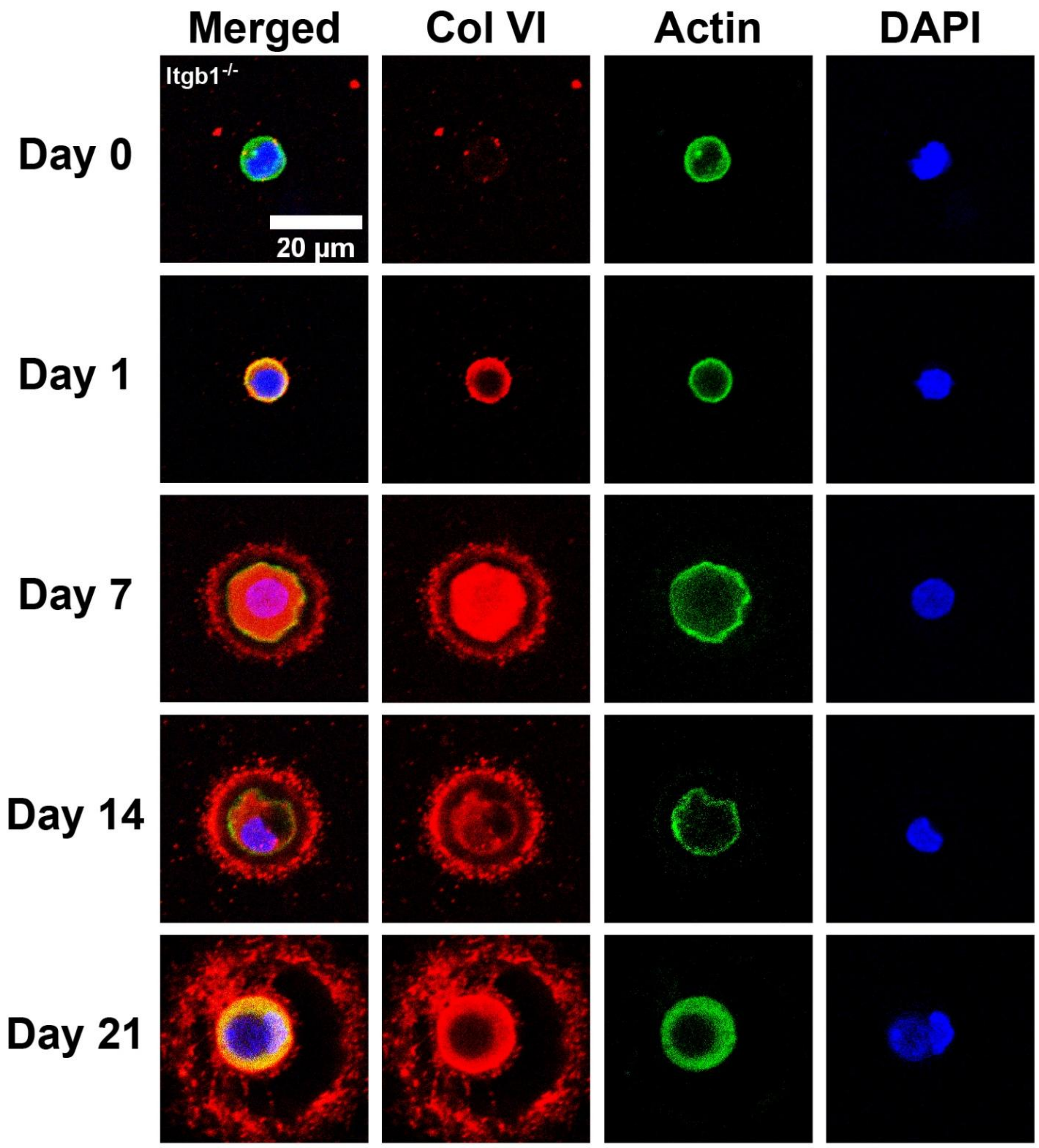


**Fig. S4. PCM of Itgb1$^{-/-}$ chondrocytes was expanded as a function of days of culture.** Blue: DAPI. Red: Col VI. Green: actin.

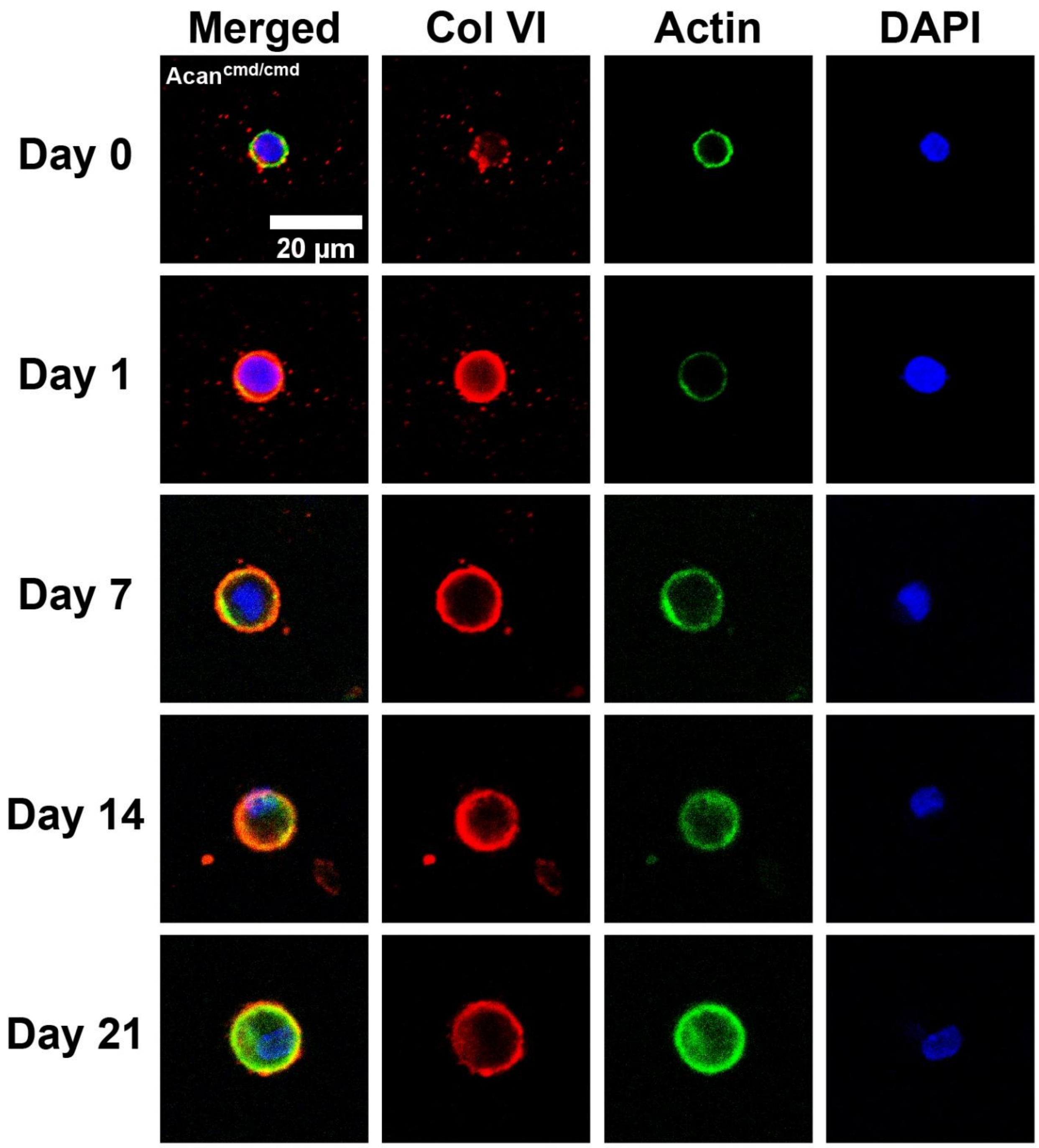


**Fig. S5. Deletion of aggrecan causes localization of PCM on the membrane of Acan$^{cmd/cmd}$ chondrocytes.** Blue: DAPI. Red: Col VI. Green: actin.

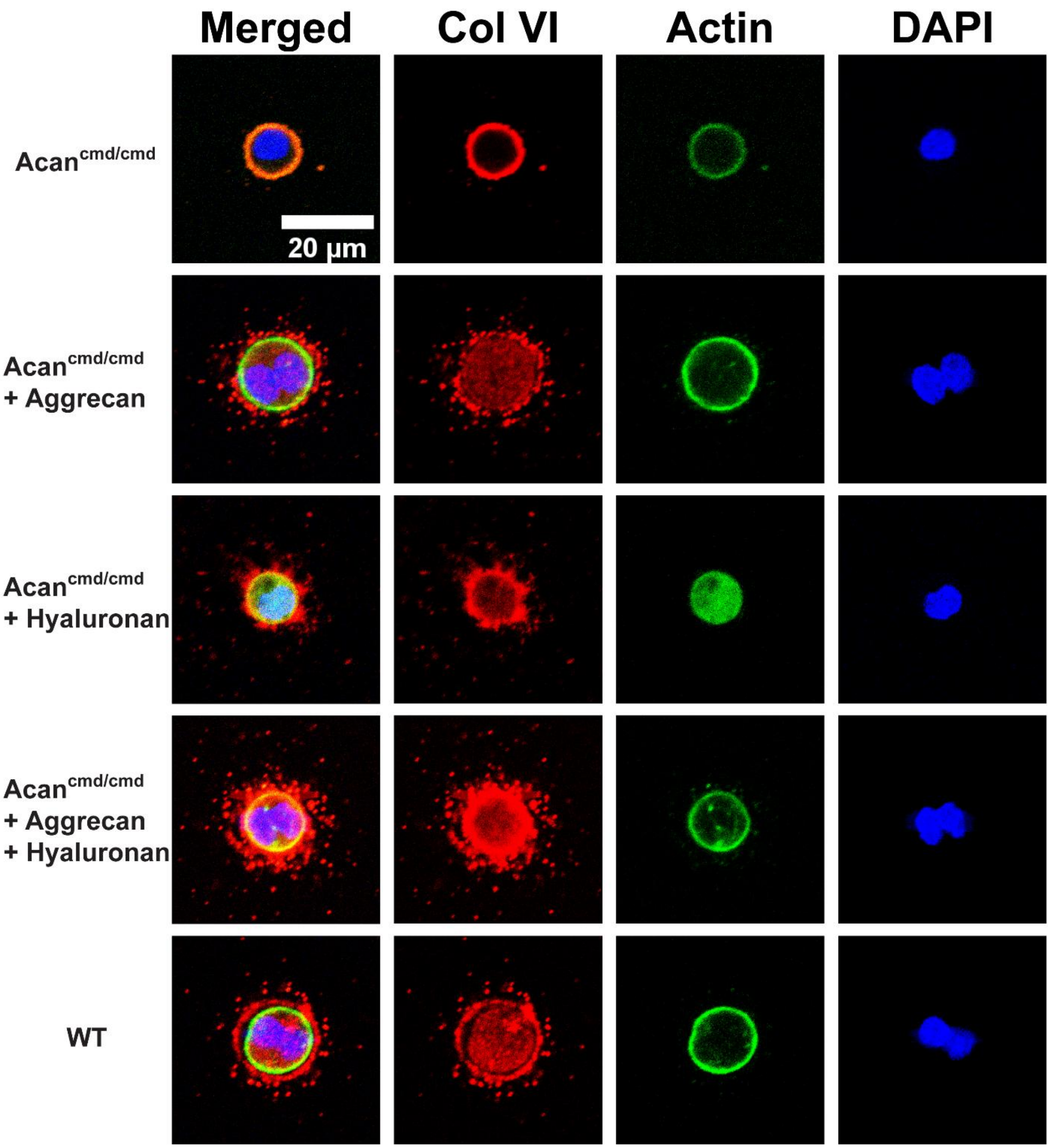


**Fig. S6. Exogeneous aggrecan and hyaluronan pushed PCM further from cell membrane of Acan$^{cmd/cmd}$ chondrocytes.** Exogenous factors were added to 3D cultured Acan$^{cmd/cmd}$ chondrocytes from day 0 to day 3. At day 4, the sample was collected and fixed. Blue: DAPI. Red: Col VI. Green: actin.

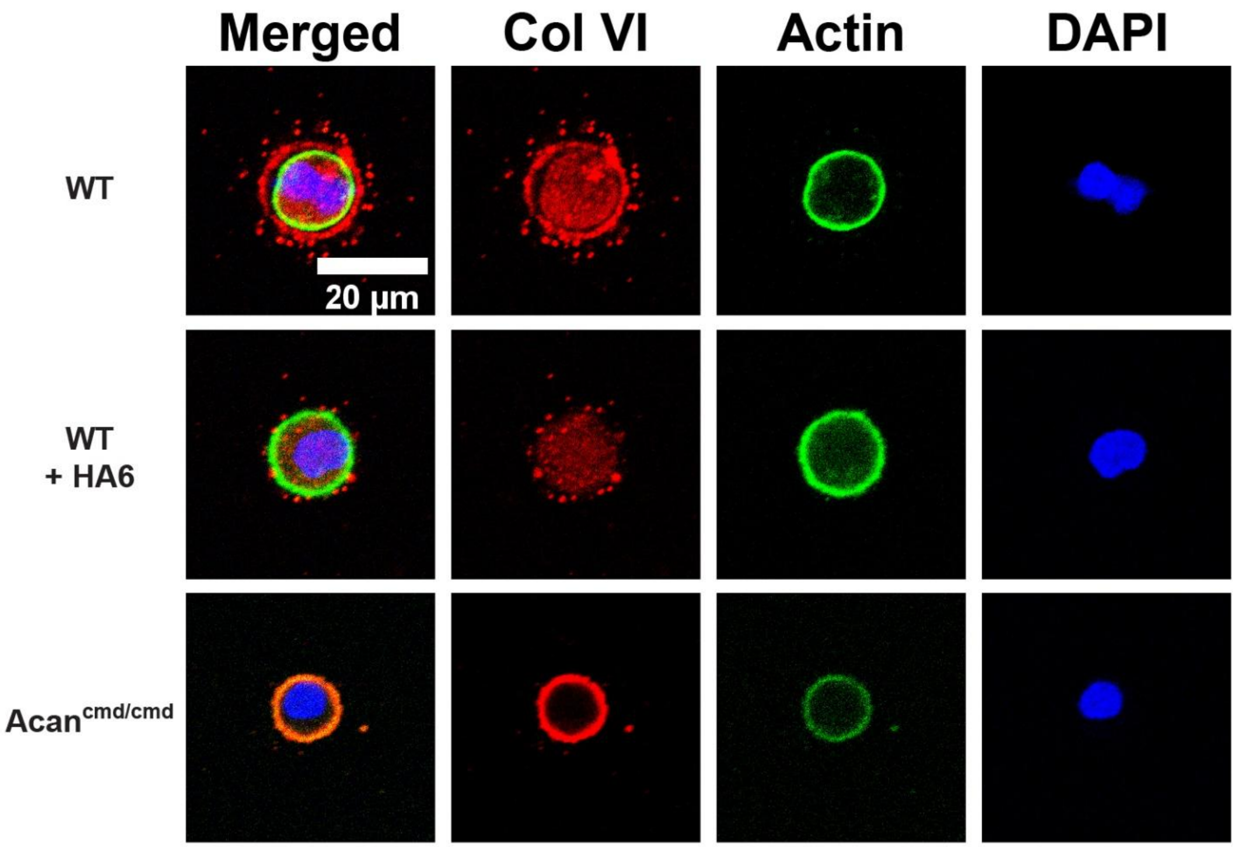


**Fig. S7. Inhibiting CD 44 and hyaluronan (HA) assembly using HA6 disrupts PCM expansion from cell membrane.** 3D cultured WT chondrocytes were treated with HA6 from day 0 to day 3. At day 4, the sample was collected and fixed. Blue: DAPI. Red: Col VI. Green: actin.

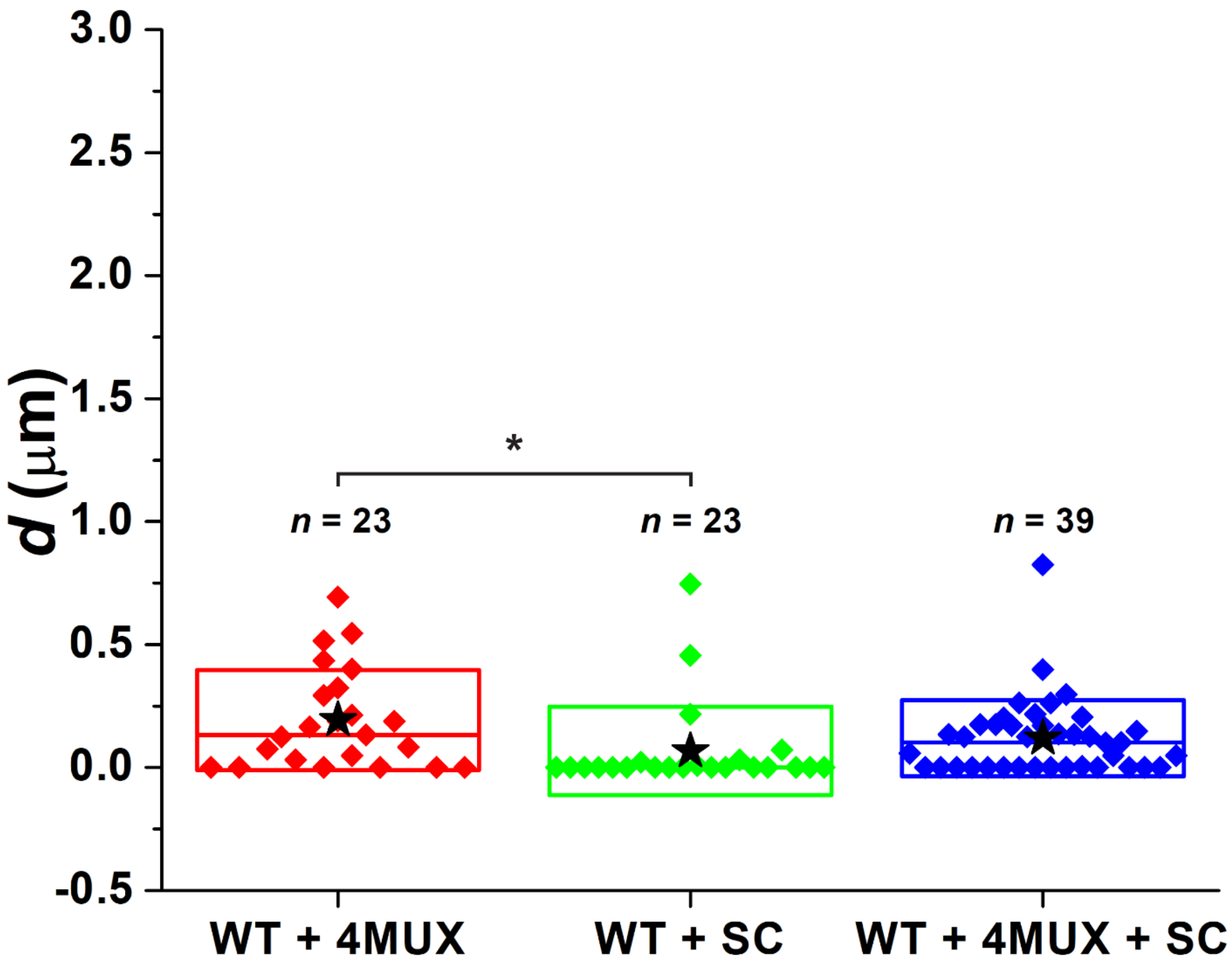


**Fig. S8.** ***d*** **of chondrocytes as a function of 4-Methylumbelliferyl-β-D-xylopyranoside (4MUX) and sodium chlorate (SC) treatment.** At day 4, the sample was collected and fixed.

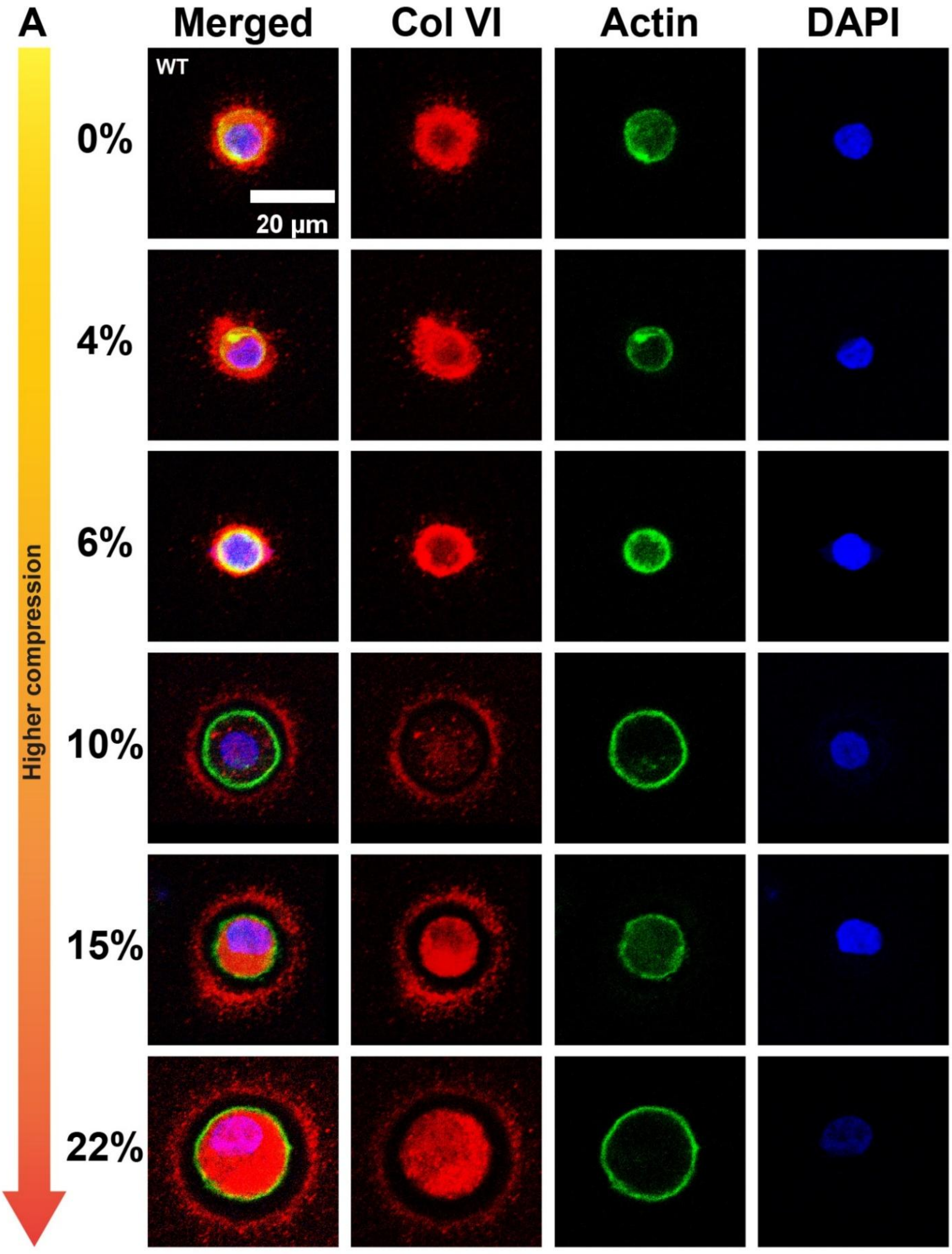
A
Merged
Col VI
Actin
DAPI
WT
0%
20 µm
4%
6%
10%
15%
22%
Higher compression

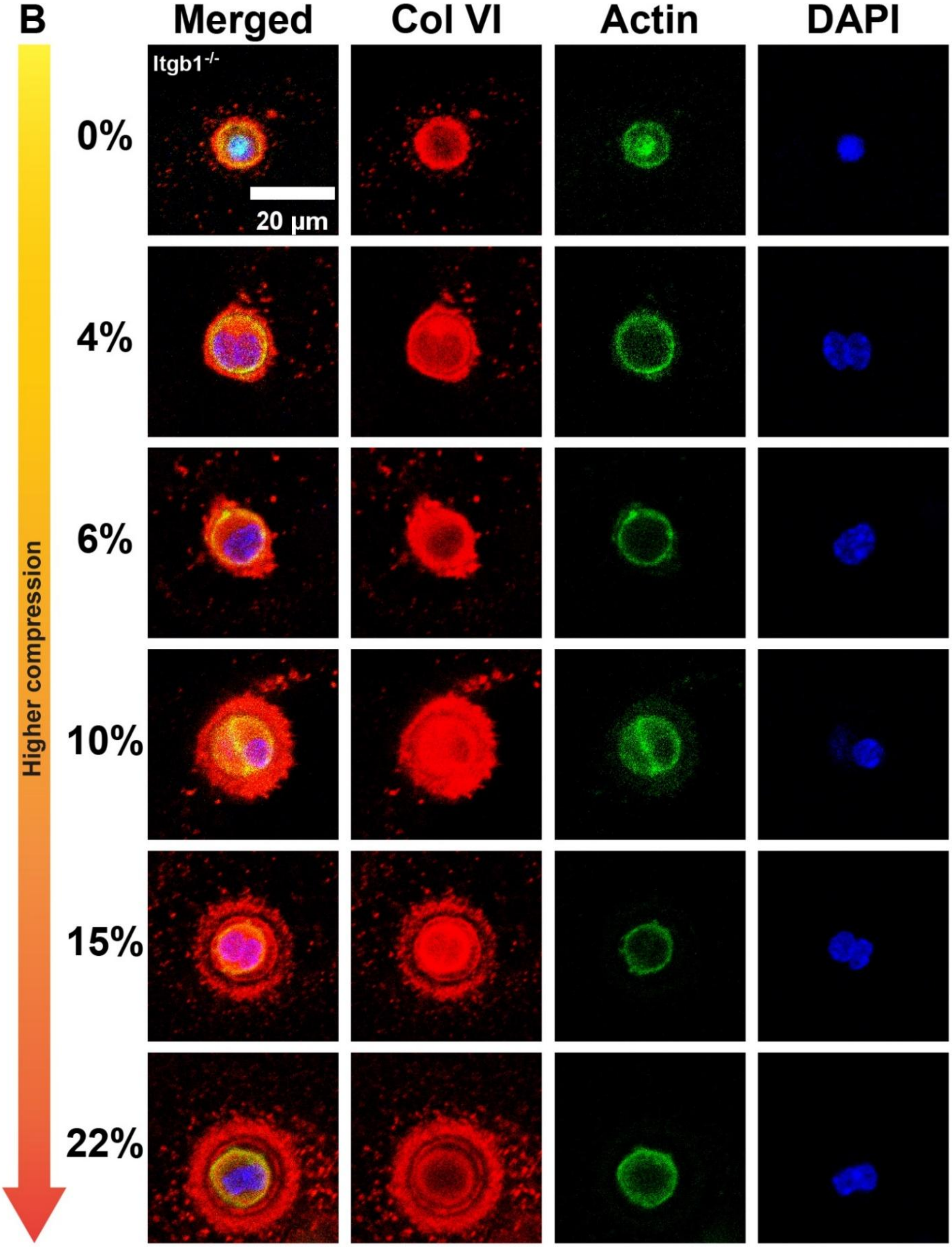
B
Merged
Col VI
Actin
DAPI
Itgb1-/-
20 µm
0%
4%
6%
10%
15%
22%
Higher compression

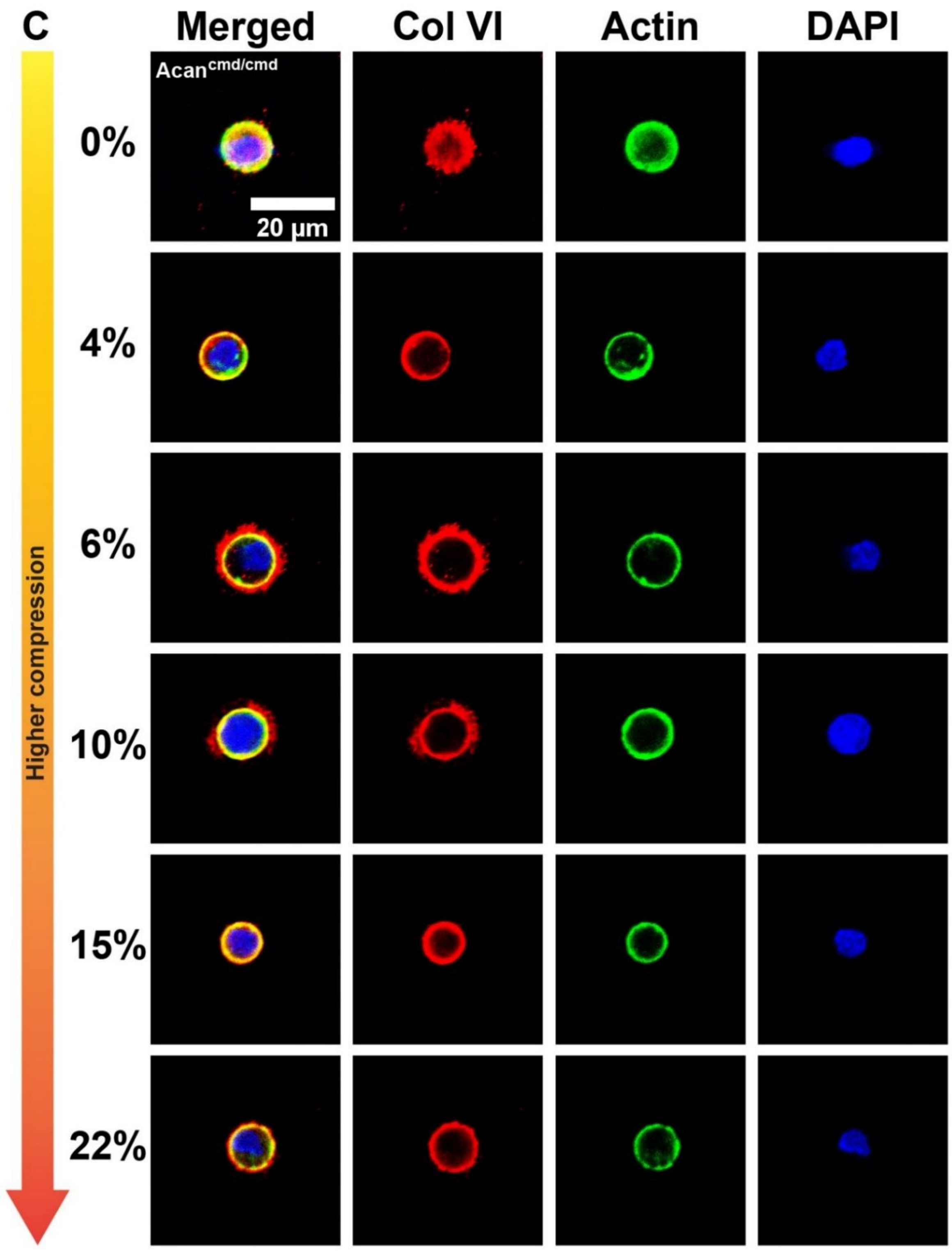


**Fig. S9. The effects of dynamic compression (1 hour/day, 1 Hz) on PCM deposition of (A) WT, (B) Itgb1$^{-/-}$ and (C) Acan$^{cmd/cmd}$ chondrocytes.** The samples were dynamically compressed at 1 Hz for 1 hour on days 1 - 3 and collected at day 4. Blue: DAPI. Red: Col VI. Green: actin.

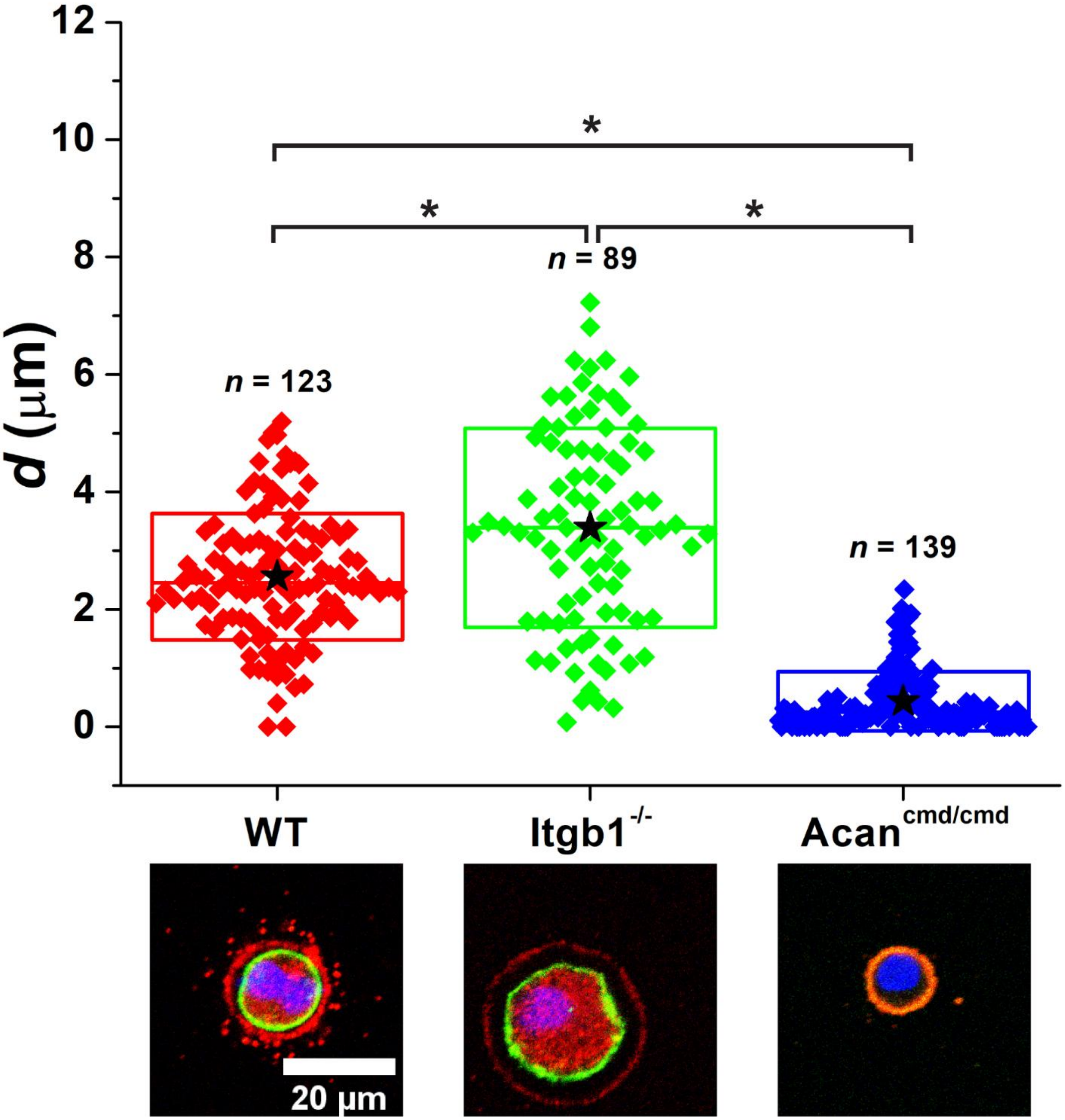


**Fig. S10. Integrin β1 is not a crucial component for PCM assembly.** At day 4, the sample was collected and fixed. Blue: DAPI. Red: Col VI. Green: actin. Star: mean values. Diamond: each data points. Top and bottom lines of the box: standard deviation. Middle line of the box: median value. *: $p < 0.05$.

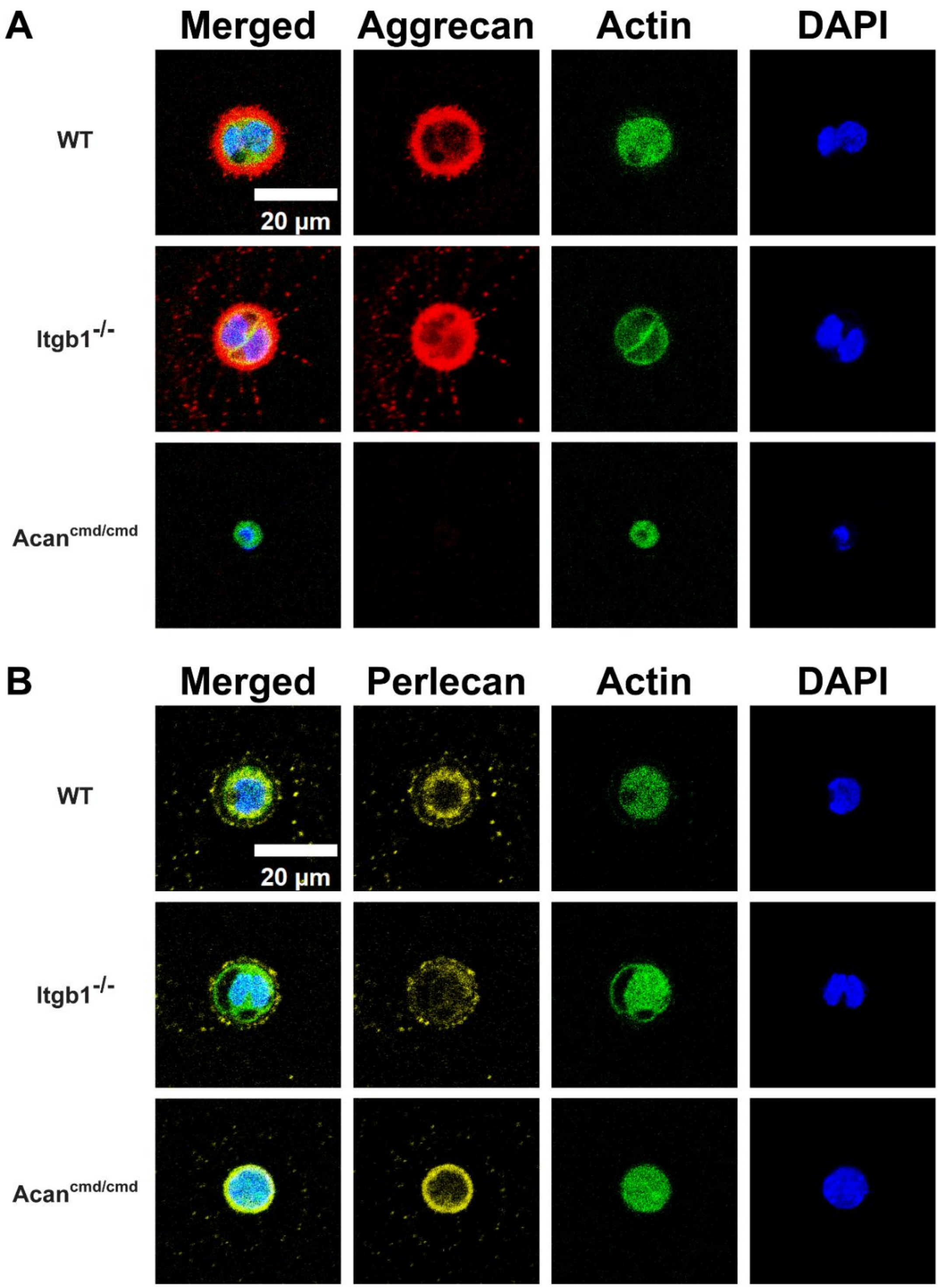


**Fig. S11. Localization of aggrecan and perlecan around chondrocytes.** (A) Aggrecan closely surrounds both WT and Itgb1-/- chondrocytes, with no gap observed between the cell membrane and the aggrecan layer. (B) A visible gap exists between perlecan and the cell membrane in both WT and Itgb1$^{-/-}$ chondrocytes, whereas perlecan is directly localized on the cell membrane of Acan$^{cmd/cmd}$ chondrocytes. At day 4, the sample was collected and fixed. Blue: DAPI. Red: aggrecan. Green: actin. Yellow: perlecan.

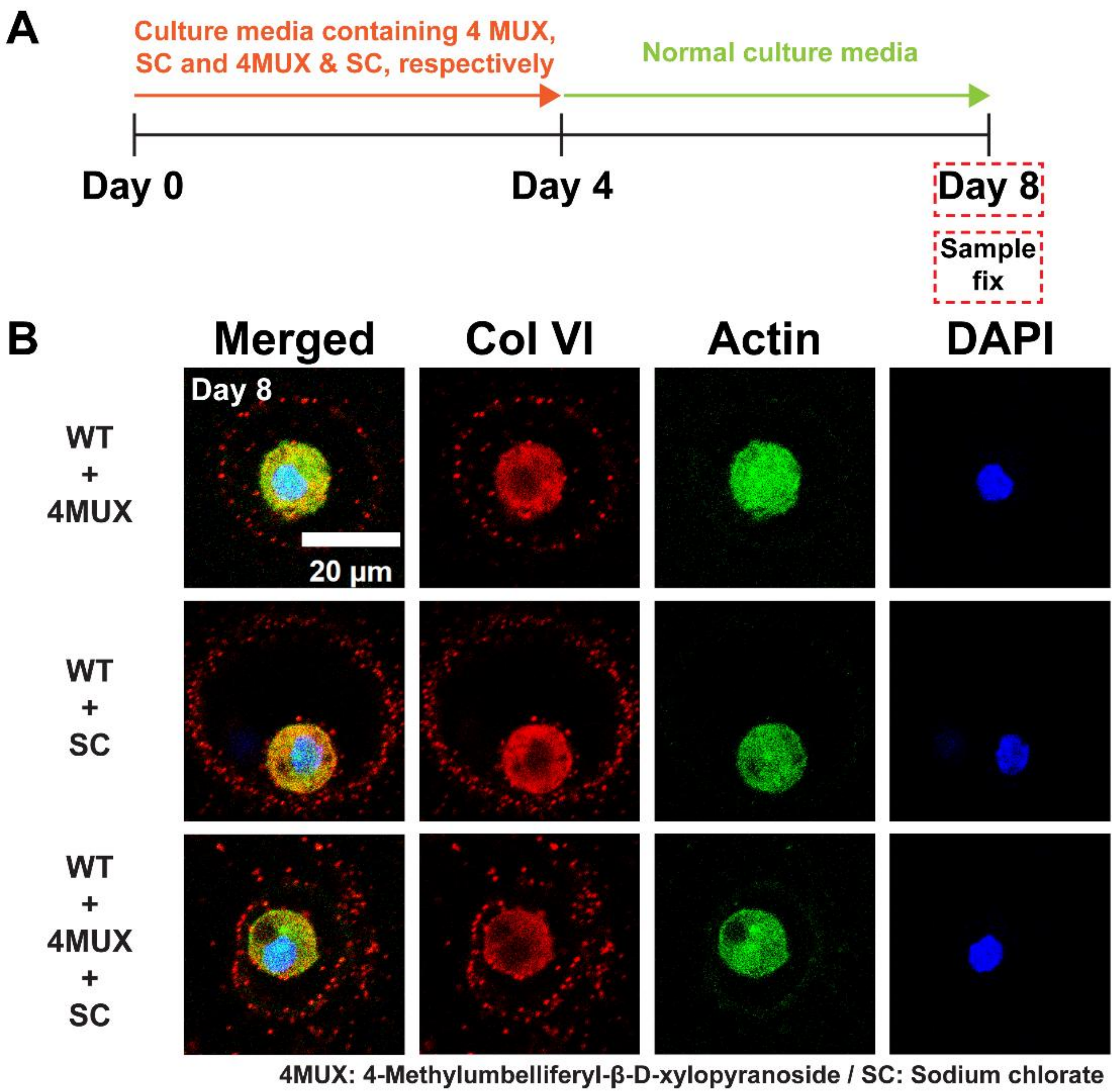


**Fig. S12. Effects of 4-Methylumbelliferyl-β-D-xylopyranoside (4MUX) and sodium chlorate (SC) on PCM expansion.** (A) WT chondrocytes were treated with 4MUX, SC, or a combination of 4MUX and SC for 4 days, followed by a wash and an additional 4-day culture in normal media. (B) After removing 4MUX and SC, abnormal expansion of PCM is observed on Day 8. Blue: DAPI. Red: Col VI. Green: actin.

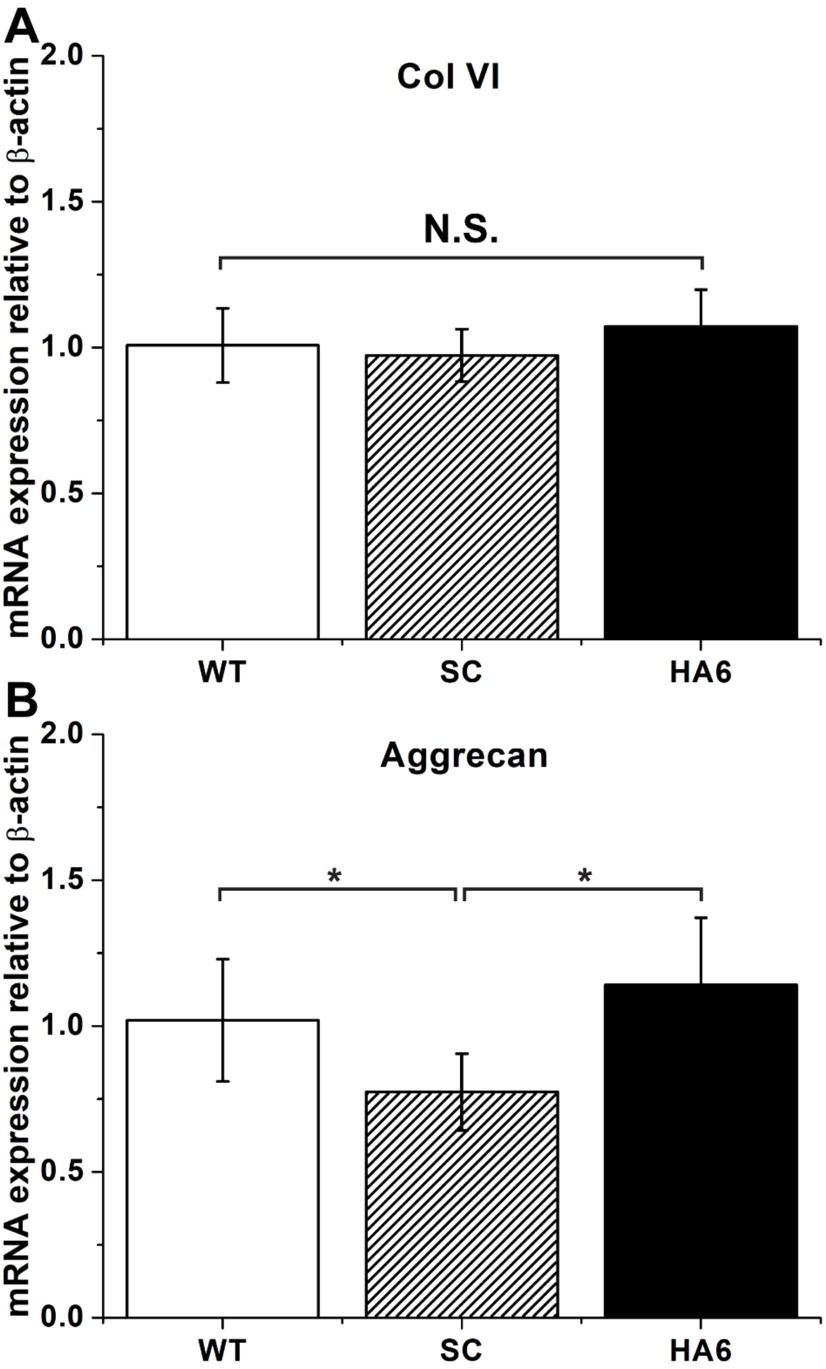


**Fig. S13. mRNA expression levels of Col VI (A) and aggrecan (B).**

## Table S1. Statistical analysis details, including p-values and methods.

| | | | | P value | Summary | Analysis |
|---|---|---|---|---|---|---|
| Figure 3 | Day 4 | vs | Day 1 | 4.0E-09 | * | One-way ANOVA |
| | Day 7 | vs | Day 1 | 0.0E+00 | * | One-way ANOVA |
| | Day 7 | vs | Day 4 | 1.1E-08 | * | One-way ANOVA |
| | Day 10 | vs | Day 1 | 0.0E+00 | * | One-way ANOVA |
| | Day 10 | vs | Day 4 | 1.1E-08 | * | One-way ANOVA |
| | Day 10 | vs | Day 7 | 1.0E+00 | N.S. | One-way ANOVA |
| | Day14 | vs | Day 1 | 0.0E+00 | * | One-way ANOVA |
| | Day14 | vs | Day 4 | 1.9E-08 | * | One-way ANOVA |
| | Day14 | vs | Day 7 | 1.0E+00 | N.S. | One-way ANOVA |
| | Day14 | vs | Day 10 | 1.0E+00 | N.S. | One-way ANOVA |
| | Day 17 | vs | Day 1 | 0.0E+00 | * | One-way ANOVA |
| | Day 17 | vs | Day 4 | 9.6E-08 | * | One-way ANOVA |
| | Day 17 | vs | Day 7 | 1.0E+00 | N.S. | One-way ANOVA |
| | Day 17 | vs | Day 10 | 9.9E-01 | N.S. | One-way ANOVA |
| | Day 17 | vs | Day14 | 1.0E+00 | N.S. | One-way ANOVA |
| | Day 21 | vs | Day 1 | 4.0E-09 | * | One-way ANOVA |
| | Day 21 | vs | Day 4 | 4.0E-05 | * | One-way ANOVA |
| | Day 21 | vs | Day 7 | 1.0E+00 | N.S. | One-way ANOVA |
| | Day 21 | vs | Day 10 | 9.9E-01 | N.S. | One-way ANOVA |
| | Day 21 | vs | Day14 | 1.0E+00 | N.S. | One-way ANOVA |
| | Day 21 | vs | Day 17 | 1.0E+00 | N.S. | One-way ANOVA |
| Figure 4 | Day 4 | vs | Day 1 | 3.4E-06 | * | One-way ANOVA |
| | Day 7 | vs | Day 1 | 2.3E-08 | * | One-way ANOVA |
| | Day 7 | vs | Day 4 | 1.1E-01 | N.S. | One-way ANOVA |
| | Day 10 | vs | Day 1 | 0.0E+00 | * | One-way ANOVA |
| | Day 10 | vs | Day 4 | 8.9E-06 | * | One-way ANOVA |
| | Day 10 | vs | Day 7 | 1.4E-01 | N.S. | One-way ANOVA |
| | Day14 | vs | Day 1 | 1.6E-08 | * | One-way ANOVA |
| | Day14 | vs | Day 4 | 2.0E-04 | * | One-way ANOVA |
| | Day14 | vs | Day 7 | 4.3E-01 | N.S. | One-way ANOVA |
| | Day14 | vs | Day 10 | 1.0E+00 | N.S. | One-way ANOVA |
| | Day 17 | vs | Day 1 | 0.0E+00 | * | One-way ANOVA |
| | Day 17 | vs | Day 4 | 0.0E+00 | * | One-way ANOVA |
| | Day 17 | vs | Day 7 | 8.7E-08 | * | One-way ANOVA |
| | Day 17 | vs | Day 10 | 4.5E-02 | * | One-way ANOVA |
| | Day 17 | vs | Day14 | 8.8E-03 | * | One-way ANOVA |
| | Day 21 | vs | Day 1 | 0.0E+00 | * | One-way ANOVA |
| | Day 21 | vs | Day 4 | 7.5E-09 | * | One-way ANOVA |
| | Day 21 | vs | Day 7 | 2.1E-08 | * | One-way ANOVA |
| | Day 21 | vs | Day 10 | 3.5E-04 | * | One-way ANOVA |
| | Day 21 | vs | Day14 | 4.3E-05 | * | One-way ANOVA |
| | Day 21 | vs | Day 17 | 6.2E-01 | N.S. | One-way ANOVA |
| Figure 5 | Day 4 | vs | Day 1 | 3.8E-01 | N.S. | One-way ANOVA |
| | Day 7 | vs | Day 1 | 1.0E+00 | N.S. | One-way ANOVA |
| | Day 7 | vs | Day 4 | 1.2E-02 | * | One-way ANOVA |
| | Day 10 | vs | Day 1 | 1.0E+00 | N.S. | One-way ANOVA |
| | Day 10 | vs | Day 4 | 8.9E-02 | N.S. | One-way ANOVA |
| | Day 10 | vs | Day 7 | 1.0E+00 | N.S. | One-way ANOVA |
| | Day14 | vs | Day 1 | 9.4E-01 | N.S. | One-way ANOVA |
| | Day14 | vs | Day 4 | 9.2E-01 | N.S. | One-way ANOVA |
| | Day14 | vs | Day 7 | 4.7E-01 | N.S. | One-way ANOVA |
| | Day14 | vs | Day 10 | 6.7E-01 | N.S. | One-way ANOVA |
| | Day 17 | vs | Day 1 | 1.0E+00 | N.S. | One-way ANOVA |
| | Day 17 | vs | Day 4 | 3.8E-01 | N.S. | One-way ANOVA |
| | Day 17 | vs | Day 7 | 1.0E+00 | N.S. | One-way ANOVA |
| | Day 17 | vs | Day 10 | 1.0E+00 | N.S. | One-way ANOVA |
| | Day 17 | vs | Day14 | 9.3E-01 | N.S. | One-way ANOVA |
| | Day 21 | vs | Day 1 | 9.0E-01 | N.S. | One-way ANOVA |
| | Day 21 | vs | Day 4 | 9.7E-01 | N.S. | One-way ANOVA |
| | Day 21 | vs | Day 7 | 3.8E-01 | N.S. | One-way ANOVA |
| | Day 21 | vs | Day 10 | 5.8E-01 | N.S. | One-way ANOVA |
| | Day 21 | vs | Day14 | 1.0E+00 | N.S. | One-way ANOVA |
| | Day 21 | vs | Day 17 | 8.9E-01 | N.S. | One-way ANOVA |

| | | | | | | |
|---|---|---|---|---|---|---|
| Figure 6 | Acan$^{cmd}$ + Aggrecan | vs | Acan$^{cmd}$ | 5.0E-08 | * | One-way ANOVA |
| | Acan$^{cmd}$ + Hyaluronan | vs | Acan$^{cmd}$ | 6.3E-04 | * | One-way ANOVA |
| | Acan$^{cmd}$ + Hyaluronan | vs | Acan$^{cmd}$ + Aggrecan | 9.7E-01 | N.S. | One-way ANOVA |
| | Acan$^{cmd}$ + Aggrecan + Hyaluronan | vs | Acan$^{cmd}$ | 0.0E+00 | * | One-way ANOVA |
| | Acan$^{cmd}$ + Aggrecan + Hyaluronan | vs | Acan$^{cmd}$ + Aggrecan | 4.4E-09 | * | One-way ANOVA |
| | Acan$^{cmd}$ + Aggrecan + Hyaluronan | vs | Acan$^{cmd}$ + Hyaluronan | 7.3E-09 | * | One-way ANOVA |
| | WT | vs | Acan$^{cmd}$ | 0.0E+00 | * | One-way ANOVA |
| | WT | vs | Acan$^{cmd}$ + Aggrecan | 0.0E+00 | * | One-way ANOVA |
| | WT | vs | Acan$^{cmd}$ + Hyaluronan | 1.2E-09 | * | One-way ANOVA |
| | WT | vs | Acan$^{cmd}$ + Aggrecan + Hyaluronan | 3.4E-04 | * | One-way ANOVA |
| Figure 7 | WT + HA6 | vs | WT | 0.0E+00 | * | One-way ANOVA |
| | Acan$^{cmd}$ | vs | WT | 0.0E+00 | * | One-way ANOVA |
| | Acan$^{cmd}$ | vs | WT + HA6 | 0.0E+00 | * | One-way ANOVA |
| Figure 9E WT | 3.5% | vs | 0.0% | 1.0E+00 | N.S. | One-way ANOVA |
| | 6.3% | vs | 0.0% | 4.4E-01 | N.S. | One-way ANOVA |
| | 6.3% | vs | 3.5% | 7.3E-01 | N.S. | One-way ANOVA |
| | 10.1% | vs | 0.0% | 6.4E-01 | N.S. | One-way ANOVA |
| | 10.1% | vs | 3.5% | 5.5E-01 | N.S. | One-way ANOVA |
| | 10.1% | vs | 6.3% | 3.7E-02 | * | One-way ANOVA |
| | 15.0% | vs | 0.0% | 6.5E-09 | * | One-way ANOVA |
| | 15.0% | vs | 3.5% | 9.3E-09 | * | One-way ANOVA |
| | 15.0% | vs | 6.3% | 4.6E-09 | * | One-way ANOVA |
| | 15.0% | vs | 10.1% | 2.4E-06 | * | One-way ANOVA |
| | 22.0% | vs | 0.0% | 8.0E-09 | * | One-way ANOVA |
| | 22.0% | vs | 3.5% | 1.1E-08 | * | One-way ANOVA |
| | 22.0% | vs | 6.3% | 5.7E-09 | * | One-way ANOVA |
| | 22.0% | vs | 10.1% | 1.2E-05 | * | One-way ANOVA |
| | 22.0% | vs | 15.0% | 1.0E+00 | N.S. | One-way ANOVA |
| Figure 9E Itgb1$^{-/-}$ | 3.5% | vs | 0.0% | 1.0E+00 | N.S. | One-way ANOVA |
| | 6.3% | vs | 0.0% | 9.3E-01 | N.S. | One-way ANOVA |
| | 6.3% | vs | 3.5% | 9.8E-01 | N.S. | One-way ANOVA |
| | 10.1% | vs | 0.0% | 1.0E+00 | N.S. | One-way ANOVA |
| | 10.1% | vs | 3.5% | 1.0E+00 | N.S. | One-way ANOVA |
| | 10.1% | vs | 6.3% | 9.9E-01 | N.S. | One-way ANOVA |
| | 15.0% | vs | 0.0% | 6.2E-01 | N.S. | One-way ANOVA |
| | 15.0% | vs | 3.5% | 4.6E-01 | N.S. | One-way ANOVA |
| | 15.0% | vs | 6.3% | 2.4E-01 | N.S. | One-way ANOVA |
| | 15.0% | vs | 10.1% | 4.6E-01 | N.S. | One-way ANOVA |
| | 22.0% | vs | 0.0% | 4.8E-02 | * | One-way ANOVA |
| | 22.0% | vs | 3.5% | 2.3E-02 | * | One-way ANOVA |
| | 22.0% | vs | 6.3% | 1.2E-02 | * | One-way ANOVA |
| | 22.0% | vs | 10.1% | 2.9E-02 | * | One-way ANOVA |
| | 22.0% | vs | 15.0% | 9.3E-01 | N.S. | One-way ANOVA |
| Figure 9E Acan$^{cmd/cmd}$ | 3.5% | vs | 0.0% | 9.5E-01 | N.S. | One-way ANOVA |
| | 6.3% | vs | 0.0% | 3.2E-01 | N.S. | One-way ANOVA |
| | 6.3% | vs | 3.5% | 9.3E-01 | N.S. | One-way ANOVA |
| | 10.1% | vs | 0.0% | 9.0E-03 | * | One-way ANOVA |
| | 10.1% | vs | 3.5% | 2.2E-01 | N.S. | One-way ANOVA |
| | 10.1% | vs | 6.3% | 7.5E-01 | N.S. | One-way ANOVA |
| | 15.0% | vs | 0.0% | 8.1E-01 | N.S. | One-way ANOVA |
| | 15.0% | vs | 3.5% | 1.0E+00 | N.S. | One-way ANOVA |
| | 15.0% | vs | 6.3% | 9.8E-01 | N.S. | One-way ANOVA |
| | 15.0% | vs | 10.1% | 2.9E-01 | N.S. | One-way ANOVA |
| | 22.0% | vs | 0.0% | 1.4E-01 | N.S. | One-way ANOVA |
| | 22.0% | vs | 3.5% | 9.2E-01 | N.S. | One-way ANOVA |
| | 22.0% | vs | 6.3% | 1.0E+00 | N.S. | One-way ANOVA |
| | 22.0% | vs | 10.1% | 5.8E-01 | N.S. | One-way ANOVA |
| | 22.0% | vs | 15.0% | 9.7E-01 | N.S. | One-way ANOVA |
| Figure S8 | WT + SC | vs | WT + 4MUX | 4.5E-02 | * | One-way ANOVA |
| | WT + 4MUX + SC | vs | WT + 4MUX | 2.5E-01 | N.S. | One-way ANOVA |
| | WT + 4MUX + SC | vs | WT + SC | 5.0E-01 | N.S. | One-way ANOVA |
| Figure S10 | Itgb1$^{-/-}$ | vs | WT | 4.1E-07 | * | One-way ANOVA |
| | Acan$^{cmd}$ | vs | WT | 0.0E+00 | * | One-way ANOVA |
| | Acan$^{cmd}$ | vs | Itgb1$^{-/-}$ | 0.0E+00 | * | One-way ANOVA |
| Figure S13A | SC | vs | WT | 7.5E-01 | N.S. | One-way ANOVA |
| | HA6 | vs | WT | 3.7E-01 | N.S. | One-way ANOVA |
| | HA6 | vs | SC | 1.1E-01 | N.S. | One-way ANOVA |
| Figure S13B | SC | vs | WT | 1.1E-02 | * | One-way ANOVA |
| | HA6 | vs | WT | 2.9E-01 | N.S. | One-way ANOVA |
| | HA6 | vs | SC | 1.6E-04 | * | One-way ANOVA |
